# Establishment and Molecular Characterization of an In Vitro Model for PARPi-Resistant Ovarian Cancer

**DOI:** 10.3390/cancers15153774

**Published:** 2023-07-25

**Authors:** Daniel Martin Klotz, Franziska Maria Schwarz, Anna Dubrovska, Kati Schuster, Mirko Theis, Alexander Krüger, Oliver Kutz, Theresa Link, Pauline Wimberger, Stephan Drukewitz, Frank Buchholz, Jürgen Thomale, Jan Dominik Kuhlmann

**Affiliations:** 1 Department of Gynecology and Obstetrics, Medical Faculty and University Hospital Carl Gustav Carus, Technische Universität Dresden, 01307 Dresden, Germany; daniel.m.klotz@ukdd.de (D.M.K.); franziska.schwarz@ukdd.de (F.M.S.); kati.schuster@ukdd.de (K.S.); oliver.kutz@ukdd.de (O.K.); theresa.link@ukdd.de (T.L.); pauline.wimberger@ukdd.de (P.W.); 2 National Center for Tumor Diseases (NCT), 01307 Dresden, Germany; German Cancer Research Center (DKFZ), 69120 Heidelberg, Germany; Faculty of Medicine, University Hospital Carl Gustav Carus, Technische Universität Dresden, 01307 Dresden, Germany; Helmholtz-Zentrum Dresden-Rossendorf (HZDR), 01307 Dresden, Germany; anna.dubrovska@ukdd.de (A.D.); mirko.theis@tu-dresden.de (M.T.); alexander.krueger@ukdd.de (A.K.); stephan.drukewitz@medizin.uni-leipzig.de (S.D.); frank.buchholz@tu-dresden.de (F.B.); 3 German Cancer Consortium (DKTK), Partner Site Dresden and German Cancer Research Center (DKFZ), 69120 Heidelberg, Germany; 4 Helmholtz-Zentrum Dresden-Rossendorf, Institute of Radiooncology-OncoRay, 01328 Dresden, Germany; 5 OncoRay-National Center for Radiation Research in Oncology, Faculty of Medicine and University Hospital Carl Gustav Carus, Technische Universität Dresden and Helmholtz-Zentrum Dresden-Rossendorf, 01307 Dresden, Germany; 6 UCC Section Medical Systems Biology, Medical Faculty and University Hospital Carl Gustav Carus, Technische Universität Dresden, 01307 Dresden, Germany; 7 Core Unit for Molecular Tumor Diagnostics (CMTD), National Center for Tumor Diseases (NCT), Partner Site Dresden, German Cancer Consortium (DKTK), Dresden, German Cancer Research Center (DKFZ), 69120 Heidelberg, Germany; 8 Institute of Human Genetics, University of Leipzig Medical Center, 04103 Leipzig, Germany; 9 Institute of Cell Biology (Cancer Research), University of Duisburg-Essen Medical School, 45147 Essen, Germany; juergen.thomale@uk-essen.de

**Keywords:** ovarian cancer, PARPi resistance, cisplatin

## Abstract

**Simple Summary:**

We established an in vitro model for PARPi-resistant ovarian cancer by long-term olaparib exposure of either *BRCA1*-proficient or *BRCA1*-deficient cell lines. We describe that PARPi-resistant cells show a broad spectrum of cross-resistance toward other clinically relevant PARPis and chemotherapeutic drugs independently of the *BRCA1*-status of the parental cell lines. Using co-culture experiments, we studied the clonal dynamics of our in vitro model and showed that PARPi-resistant cells have a proliferative disadvantage over PARPi-sensitive cells under drug-free conditions, while they rapidly gained clonal dominance under selection pressure with olaparib, which can be blocked by pharmacological inhibition of ABC-transporter proteins. Conclusively, we present a well-characterized in vitro model of PARPi resistance that could be useful in dissecting mechanisms of PARPi resistance derived from a *BRCA1*-proficient or *BRCA1*-deficient background. Furthermore, our model will allow studying how experimental therapeutic drugs, such as novel PARPi-sensitizers, affect the clonal dynamics of PARPi-resistant cells.

**Abstract:**

Overcoming PARPi resistance is a high clinical priority. We established and characterized comparative in vitro models of acquired PARPi resistance, derived from either a *BRCA1*-proficient or *BRCA1*-deficient isogenic background by long-term exposure to olaparib. While parental cell lines already exhibited a certain level of intrinsic activity of multidrug resistance (MDR) proteins, resulting PARPi-resistant cells from both models further converted toward MDR. In both models, the PARPi-resistant phenotype was shaped by (i) cross-resistance to other PARPis (ii) impaired susceptibility toward the formation of DNA-platinum adducts upon exposure to cisplatin, which could be reverted by the drug efflux inhibitors verapamil or diphenhydramine, and (iii) reduced PARP-trapping activity. However, the signature and activity of ABC-transporter expression and the cross-resistance spectra to other chemotherapeutic drugs considerably diverged between the *BRCA1*-proficient vs. *BRCA1-*deficient models. Using dual-fluorescence co-culture experiments, we observed that PARPi-resistant cells had a competitive disadvantage over PARPi-sensitive cells in a drug-free medium. However, they rapidly gained clonal dominance under olaparib selection pressure, which could be mitigated by the MRP1 inhibitor MK-751. Conclusively, we present a well-characterized in vitro model, which could be instrumental in dissecting mechanisms of PARPi resistance from HR-proficient vs. HR-deficient background and in studying clonal dynamics of PARPi-resistant cells in response to experimental drugs, such as novel olaparib-sensitizers.

## 1. Introduction

Ovarian cancer is the leading cause of death among gynecological malignancies [1]. Due to the lack of early symptoms, about 75% of the patients are diagnosed at advanced stages. Standard treatment of advanced ovarian cancer consists of surgical debulking, aiming at macroscopic complete tumor resection, and platinum/paclitaxel-based chemotherapy, followed by maintenance treatment with antiangiogenic bevacizumab [2,3,4].

In ovarian cancer patients with homologous recombination (HR) deficiency (HRD), defined by either a pathogenic breast cancer 1/2, early onset (*BRCA1/2*) mutation, and/or genomic instability, a combination of bevacizumab with the poly ADP ribose polymerase inhibitor (PARPi) olaparib has been approved as maintenance therapy after response to first-line platinum-based chemotherapy [5]. Moreover, olaparib monotreatment as maintenance treatment is approved for patients with a pathogenic *BRCA1/2* mutation after response to first-line chemotherapy [6]. The PARPi niraparib is used as maintenance therapy without bevacizumab after response to first-line platinum-based treatment, independently of the HRD or *BRCA1/2* mutational status [7]. In the case of disease recurrence of platinum-sensitive high-grade epithelial ovarian cancer, the PARPi olaparib, niraparib, and rucaparib are approved as maintenance therapy after response to second-line platinum-based chemotherapy, independently of the *BRCA*1/2 mutational status [8,9,10,11]. Nonetheless, the majority of patients with recurrent disease will develop resistance toward PARPi, resulting in poor overall prognosis of ovarian cancer.

A more detailed understanding of the molecular mechanisms of PARPi resistance is urgently needed in order to design targeted therapy approaches for PARPi-resistant patients, which may improve their prognosis upon recurrent disease. Previous studies have defined basic hallmarks of PARPi resistance. Among other mechanisms, these include increased drug efflux, decreased PARP1 trapping activity, restoration of HR, or increased replication fork stability [12]. It was shown that HRD increases sensitivity to platinum and PARPi, explaining why the clinical response to both drugs is closely related in ovarian cancer patients [13]. However, not all platinum-sensitive tumors respond to PARPi [13], whereas 40% of tumors with acquired PARPi resistance retain platinum sensitivity [14].

There is an ongoing pre-clinical effort to discover novel regulators of PARPi response based on various in vitro models of PARPi resistance [15,16,17,18]. These approaches provide insight into resistance pathways, which could be translated into innovative therapeutic targets. However, for the design of those models, numerous experimental variables need to be considered, such as (i) the genetic background of used cell lines (ii) the protocol for PARPi exposure, and (iii) the analytic resolution of the established model (cell population level vs. single cell) [15,16,17,18]. Several in vitro ovarian cancer models of PARPi resistance have been described [17,19,20,21] with considerably varying experimental frameworks. Therefore, critical assessment is highly important when comparing and interpreting results of these models. While in the majority of reports, PARPi-resistant cells have successfully been established by long-term PARPi exposure, one study reported that olaparib exposure is unlikely to produce an acquired resistance phenotype [22], indicating that in vitro models of PARPi resistance are neither interchangeable nor generalizable.

Patients without HRD and *BRCA1/2* mutation can be treated with niraparib after response to platinum-based chemotherapy in the first-line or in the recurrent setting [7,10]. However, PARPi resistance can also arise in these patients. With the exception of one study [23], it has never been studied in detail how the HR background of a cancer cell may govern the phenotype of PARPi resistance. Therefore, the objective of the present study was to create two isogenic in vitro models of PARPi-resistant ovarian cancer derived from a *BRCA1*-wildtype (HR-proficient) vs. *BRCA1*-mutated (HR-deficient) genetic background. We subsequently characterized both models with regard to (i) the spectrum of cross-resistance with a focus on the molecular response to platinum and the activity of multidrug resistance (MDR) proteins (ii) the clonal dynamics and (iii) PARP-trapping activity.

## 2. Materials and Methods

### 2.1. Cell Culture

UWB1.289 (CRL-2945™; ATCC, Manassas, VA, USA) and their olaparib-resistant subline (Olres-UWB1.289) were cultured in MEGM medium mixed 1:1 with RPMI-1640 and supplemented with 3% fetal calf serum (Sigma-Aldrich, Darmstadt, Germany; #F7524, Lot BCCB7352) as well as 1% penicillin-streptomycin (Gibco™, Life Technologies, Carlsbad, CA, USA; #15140-122). UWB1.289+BRCA1 (CRL-2946™; ATCC, Manassas, VA, USA) and their olaparib-resistant subline (Olres-UWB1.289+BRCA1) were cultured in the same medium as the UWB1.289 cell line, except for an additional supplementation with 0.2 mg/mL Geneticin^®^ (Gibco™, Life Technologies; #10131-035). Igrov-1 and the cisplatin-resistant CPres-Igrov-1 cells were cultured in RPMI-1640+GlutaMAX™-I (Gibco™; #61870-010) supplemented with 10% FCS and 1% penicillin streptomycin. HEK-293 cells were cultured in DMEM-F12 (Gibco™; 11320-074) supplemented with 10% FCS, 1% L-glutamine (Gibco™, Life Technologies, #250030081) and 1% penicillin streptomycin. All cell lines were cultured at 37 °C with 5% CO_2_. Mycoplasma testing by PCR was performed regularly by a commercial in-house service.

### 2.2. Drugs

Olaparib (AZD2281; #S1060), niraparib (MK-4827; #S2741), rucaparib (#AG-014699) veliparib (ABT-888; #S1004), talazoparib (BMN 673; #S7048), phosphate (#S1098), verapamil (CP-16533-1) HCl (#S4202), SN-38 (NK012; #S4908) and MK-571 (#S8126) were purchased from Selleckchem (Munich, Germany). Cisplatin (1 mg/mL), doxorubicin, paclitaxel (6 mg/mL), and topotecan (1 mg/mL) were purchased from Accord Healthcare GmbH (Munich, Germany). Diphenhydramine-hydrochloride (DIPH; D3630-5G), methyl methanesulfonate (MMS; #129925-5G), and treosulfane (Trecondi; #SML1252) were purchased from Sigma Aldrich. Pegylated liposomal doxorubicin (Caelyx) was purchased from Elblandkliniken Stiftung & Co. KG (Riesa, Germany).

### 2.3. Fluorometric Cell Viability Assay (48-h Treatment)

Cell viability following 48 h drug treatment of human cancer cell lines was assessed using the fluorometric CellTiter-Blue^®^ Cell Viability Assay (Promega, Fitchburg, MA, USA) according to the manufacturer’s instructions and as described previously [24]. Briefly, cancer cells were seeded at a density of 10,000 cells/well in a 96-well plate and cultured in standard medium for 24 h to allow adherence. Subsequent drug treatment was performed for 48 h. Finally, cell viability was assessed using a fluorescence reader (Infinite M200, Tecan, Männedorf, Switzerland, Software Magellan Version 7.2). The data were visualized and statistically analyzed using Prism Version 9.0 (GraphPad Software San Diego, CA, USA). Resulting dose-response curves were compared with the nested *t*-test, and IC_50_ values were determined by non-linear regression of normalized drug response. 

### 2.4. Photometric Cell Viability Assay (6-Day Treatment)

Cell viability following 6 d drug treatment of human cancer cell lines was assessed as described previously [24]. Briefly, cells were seeded on 6-well plates and cultured in standard medium for 24 h to allow adherence. Subsequent drug treatment was performed for 6 d with medium containing the respective drug, which was renewed every 72 h. 7 d after initial seeding of the cells, the cells were stained with 0.1% crystal violet solution, dried for 24 h, the cell-bound violet stain was dissolved by 10% acetic acid and, finally, the dissolved violet stain was photometrically quantified using the Tecan Reader (Infinite M200, Männedorf, Switzerland, Software Magellan Version 7.2) for viability readouts. The data were visualized and statistically analyzed using Prism Version 9.0 (GraphPad Software, CA, USA). Resulting dose-response curves were compared with the nested *t*-test, and IC_50_ values were determined by non-linear regression of normalized drug response. 

### 2.5. Cell Cycle

For cell cycle analysis, cell lines were treated with olaparib for 72 h, fixed for 30 min on ice using 4% PFA (16% FA; Electron Microscopy Sciences, Hatfield, PA, USA; 15710) diluted in DPBS (Gibco™; Life Technologies; 15190-326) and permeabilized for 30 min at RT by using 10× Intracellular Staining Perm Wash Buffer (BioLegend, San Diego, CA, USA; 421002) diluted 1:10 in DPBS. The cells were stained with 1 µg/µL DAPI (PanReac AppliChem; A1001,0010) in Cell staining buffer (BioLegend; 420201). The samples were measured on the BD LSRFortessa™ Cell Analyzer (BD Biosciences; Becton, Dickinson, and Company, Franklin Lakes, NJ, USA) using the BD FACSDiva™ Software version 9.0.1 (BD Biosciences; Becton, Dickinson, and Company). The data were analyzed using FlowJo version 10.8.1 and visualized by Prism Version 9.0 (GraphPad Software, CA, USA).

### 2.6. RNA isolation and Whole Transcriptome Sequencing

For Whole-Transcriptome Sequencing (RNA-Seq), 2.3 × 10^6^ cells (UWB1.289), 1.7 × 10^6^ cells (Olres-UWB1.289), 3.76 × 10^6^ cells (UWB1.289+BRCA1), and 2.75 × 10^6^ cells (Olres-UWB1.289+BRCA1) were harvested from T-75 flasks grown for 6 d (UWB1.289 and Olres-UWB1.289) or 8 d (UWB1.289+BRCA1 and Olres-UWB1.289+BRCA1) in drug-free medium. The RNA extraction was performed using the miRNeasy Mini Kit (Qiagen, Hilden, Germany; #74104). On-column DNA digestion was included in this protocol in order to remove residual contaminating genomic DNA. All experiments were performed in duplicates and were assayed according to the manufacturer’s instructions. For library preparation, we used the TruSeq Stranded mRNA Library Prep Kit (Illumina, San Diego, CA, USA) according to the manufacturer’s protocol, starting with 1 µg total RNA. All barcoded libraries were pooled and sequenced 2 × 75 bp paired-end on an Illumina NextSeq500 platform to obtain a minimum of 10 Mio reads per sample. Reads were trimmed using trimmomatic [25] and aligned using STAR [26]. Read counts were extracted from the alignments using featureCounts method of the Rsubread package [27]. Genes with multiple testing adjusted *p*-values (padj from DESeq2) < 0.05 were considered differentially expressed genes, including those genes with a |log_2_FC| < 1.

### 2.7. Immunofluorescence Staining

For immunofluorescence staining of vimentin and E-cadherin, cells were seeded in a 48-well plate on a cover slip and stained with either vimentin rabbit monoclonal antibody (Clone D21H3; Cell Signaling Technologies, Leiden, The Netherlands; #5741) and goat anti-rabbit IgG (H+L) Alexa Fluor™ 633 (Invitrogen, Thermo Fisher Scientific, Waltham, MA, USA; #A21071; 1:500) or E-cadherin rabbit monoclonal Ab (Clone 24D10; Cell Signaling Technologies; #3195; 1:200) and goat anti rabbit Alexa Fluor 488 (Invitrogen; #A11034; 1:500). Counterstaining of cells was performed with DAPI (PanReac AppliChem; #A1001,0010; 1 µg/mL).

### 2.8. DNA Platination Assay

UWB1.289, Olres-UWB1.289, UWB1.289+BRCA1, Olres-UWB1.289+BRCA1, Igrov-1, and CPres-Igrov-1 were plated in 2 wells of a 6-well plate per treatment and timepoint. The cells were treated for 3 h with 20 µg/mL cisplatin 72 h after seeding (all UWB-derived cell lines) or 24 h after seeding (Igrov-1 and Cpres-Igrov-1). Treatment with diphenhydramine (DIPH) and 50 µM Verapamil was started 30 min before cisplatin treatment. The cells were harvested, counted, and 10,000 cells suspended in DPBS were transferred to SuperfrostPlus™ Gold Adhesion Microscope Slides (Epredia, Braunschweig, Germany; #K5800AMNZ72). Staining and analysis of the cells were performed as described previously [28,29]. Briefly, cytospots were fixed in Methanol (Honeywell/Riedel-de Haën, Charlotte, North Carolina, USA; #10191438) at −20 °C for 30 min, denatured using 60% 70 mM NaOH/140 mM NaCl, 40% methanol (*v*/*v*) for 5 min at 4 °C and treated with 250 µg/µL pepsin (ThermoFisher, Waltham, MA, USA; #1.07192.0001, 10 FIP-U/mg) followed by 250 µg/µL proteinase K solution (ThermoFisher, #17916; 30 U/mg) for 5 min at 37 °C. Subsequently, cytospots were blocked with 5% skim milk for 30 min at room temperature, stained with primary antibody rat-anti-platinum-(GpG) adduct [Pt-(GpG)] in DNA; R-C18, 0.05 µg/mL overnight at 4 °C, with 1:400 secondary antibody Rabbit IgG anti-Rat IgG (H+L)-Cy3, MinX none (Dianova, Jackson ImmunoResearch Europe Ltd., Ely, UK; #312-165-003) for 1 h at 37 °C and counterstained with 1 µg/µL DAPI (PanReac AppliChem; #A1001,0010) for 30 min at room temperature. The slides were mounted with Mowiol and kept at 4 °C until measurement. For analysis, DAPI- and Cy3-derived signals were integrated and measured separately for individual cell nuclei using a microscope-coupled digital image analysis system (Zeiss Axioplan; ACAS 6.0 Image Analysis System, Ahrens Electronics, Bargteheide, Germany). Antibody-derived fluorescence signals were normalized to the corresponding DNA content of the same nucleus and expressed as arbitrary fluorescence units (AFU). Values were calculated as means of >100 measured cells per sample and means of at least 2 biological replicates were visualized using Prism Version 9.01 (GraphPad Software).

### 2.9. PARP-Trapping Assay

UWB1.289 cells, UWB1.289+BRCA1 cells, and their olaparib-resistant sublines were grown to 80–90% subconfluency and were treated with the respective PARPi (olaparib, niraparib, rucaparib, veliparib, talazoparib) alone or in combination with the alkylating agent methanesulfonate (MMS) for 1 h and fractionated using the Subcellular Fractionation kit (Thermo Scientific™, 78840). The protein content was measured using a BCA assay, and the amount of PARP1 in the chromatin bound and nuclear soluble fraction was analyzed by Western blot. For this, 14 µg per sample was subjected to a NuPAGE 4–12% Bis-Tris protein gel and transferred onto nitrocellulose (NC) membranes (Amersham™ Protran™ Premium 0.45 µm NC, GE Healthcare Life science, Chalfont St Giles, UK). Subsequently, (dissected) NC-membranes were incubated with PARP1 (rabbit; Cell Signaling Technologies, #9542S; 1:3000), Histone H3 (D1H2) XP^®^ (rabbit; Cell Signaling Technologies, #4499; 1:25000) and DNA Topoisomerase I (mouse; BDPharmigen™, #556597; 1:30000) antibodies. Membranes were incubated for detection with secondary antibodies, raised against rabbit (anti-rabbit IgG, HRP linked; Cell Signaling Technology; #7074,) or mouse (Peroxidase-conjugated AffiniPure Goat anti-Mouse IgG, HRP linked; Jackson ImmunoResearch; #115-035-003). Detection was performed with Amersham™ ECL™ Prime Western Blotting Detection reagent (Cytiva, Freiburg, Germany; #RPN2232). The quantification was performed using the Fiji Software, and results were visualized by Prism 9.0 (GraphPad Software).

### 2.10. MDR Assay

The multidrug resistance (MDR) phenotype of the Olres-UWB1.289 and Olres-UWB1.289+BRCA1 cells, as well as their respective controls, was assessed using the EFLUXX-ID^®^ Green Multidrug Resistance Assay kit (Enzo Life Sciences; ENZ-51029-K100) according to the manufacturer’s recommendation. Briefly, the cells were cultured for 7 days and one day before the treatment, the medium of the cells was exchanged to phenol red-free medium consisting of 50% MEBM™ Mammary Epithelial Cell Growth Basal Medium, Phenol Red Free (Lonza; CC3153) supplemented with MEGM^TM^ Mammary Epithelial Cell Growth Medium SingleQuots^TM^ Kit (Lonza; CC4136) except gentamycin and 50% RPMI-1640 without phenol red (Gibco™, ThermoFisher Scientific; 11835-105) supplemented with 10 mM HEPES (1M; Gibco™, Life Technologies; 15630-056) and 1 mM sodium pyruvate (100 mM (100×); Gibco™, Life Technologies; 11360-039). The mixed medium was supplemented with 3% fetal calf serum (Sigma-Aldrich; F7524) as well as 1% Penicillin-Streptomycin (Gibco™, Life Technologies; 15140-122). The assay was performed in the phenol red-free medium in triplicates, and the cells were incubated either in the MDR1 (*ABCB1*) inhibitor verapamil, the MRP1 (*ABCC1*) inhibitor MK-571, the BCRP (*ABCG2*) inhibitor novobiocin or DMSO as a control for 5 min. Subsequently, the samples are stained with Efluxx-ID^®^ green for 30 min at 37 °C and counterstained with propidium iodide (PI) to exclude dead cells. The mean fluorescence intensity (MFI) was measured per sample using flow cytometry (BD LSRFortessa™ Cell Analyzer (BD Biosciences; Becton, Dickinson, and Company) using the BD FACSDiva™ Software version 9.0.1 (BD Biosciences; Becton, Dickinson, and Company) and FlowJo version 10.8.1 (BD Biosciences; Becton, Dickinson, and Company). If the difference between the MFI values from 3 technical replicates was <10%, the mean of the three MFIs (F) was used to calculate the Multidrug-resistant activity factor (MAF) per transporter:

$${MAF}_{transporter}=100\times \frac{F_{treated}-F_{untreated}}{F_{treated}}$$

A MAF score of >20 indicated a respective ABC-transporter activity level, which was compatible with a multidrug resistance phenotype. The MAF scores from three biological replicates were visualized using Prism 9.0 (GraphPad).

### 2.11. Proliferation Assay

UWB1.289, Olres-UWB1.289, UWB1.289+BRCA1, Olres-UWB1.289+BRCA1 cells were seeded with a density of 200,000 cells for each cell line into T25 flasks and grown for 8 d. Each day, the number of cells was counted on a LunaII™ automated cell counter (Logos Biosystems, Dongan-gu Anyang-si, Gyeaonggi-do, South Korea) using Erythrosin B stain (Logos Biosystems; #L13002) to exclude dead cells. The cell number was depicted on a logarithmic scale.

### 2.12. Generation of Color-Coded Cell Lines by Lentiviral Transduction

Color-coded UWB1.289, UWB1.289+BRCA1, as well as Olres-UWB1.289 and Olres-UWB1.289+BRCA1, cells were generated using the pWPXL-EGFP (Addgene #12257) and -tdTomato plasmids and the packaging vectors pMD2.G (Addgene #12259) and psPAX2 (Addgene #12260) as adapted from a previous study [30]. To amplify the plasmids, One Shot TOP10 *E. coli* were transformed using a heat shock of 30 s at 42 °C after 30 min incubation on ice. The *E. coli* were incubated for 1 h at 37 °C with S.O.C medium before plating them on an Agar plate and growth overnight. The plasmids were purified using the Qiagen^®^ Plasmid Maxi kit (Qiagen, Hilden, Germany; 12163) according to the manufacturer’s recommendation. Lentiviral transduction was performed as described previously [31]. Briefly, HEK293 cells were seeded to 10 cm dishes 24 h prior to transfection with 12 µg psPAX2 (Addgene #12260), 6 µg pMD2.G (Addgene #12259), and 12 µg pWPXL (Addgene #12257) with the respective fluorophore sequence (eGFP or tdTomato) by calcium phosphate precipitation. The produced virions were filtered and transferred to the medium of approx. 400,000 UWB1.289, Olres-UWB1.289, UWB1.289+BRCA1, and Olres-UWB1.289+BRCA1 cells, plated 48 h prior to transduction to 10 cm dishes to generate UWB1.289±BRCA1-pWPXL-EGFP and UWB1.289±BRCA1-pWPXL-tdTomato, as well as Olres-UWB1.289±BRCA1-pWPXL-EGFP and Olres-UWB1.289±BRCA1-pWPXL-tdTomato cells. Cells expressing eGFP or tdTomato were sorted on the BD FACSAria™ Fusion Cell Sorter (BD Biosciences; Becton, Dickinson, and Company) at the CMCB Core Facility using a 100 µm nozzle and the BD FACSDiva™ Software version 9.0.1.

### 2.13. Cell Competition Assay

To analyze clonal competition between PARPi-sensitive vs. -resistant cell lines, 100,000 UWB1.289-pWPXL-tdTomato cells were mixed with 100,000 Olres-UWB1.289-pWPXL-EGFP cells (day 0) and cultured in a T25 flask. The next day, the cells were treated with 4 µM Olaparib or 1.25 µg/mL cisplatin (day 1). The culture composition was analyzed by flow cytometry on day 0, day 3, and day 7. For the UWB1.289+BRCA1 model, 75,000 UWB1.289+BRCA1-pWPXL-tdTomato cells were mixed with 75,000 Olres-UWB1.289+BRCA1-pWPXL-EGFP cells (d0). The treatment was performed with 50 µM Olaparib or 2.5 µg/mL cisplatin on day 1, and the culture composition was analyzed by flow cytometry on day 0, day 3, day 7, day 10, and day 14. The samples were measured on the BD LSRFortessa™ Cell Analyzer (BD Biosciences; Becton, Dickinson, and Company) using the BD FACSDiva™ Software version 9.0.1 (BD Biosciences; Becton, Dickinson, and Company). The data were analyzed using FlowJo version 10.8.1 and visualized by Prism Version 9.0 (GraphPad Software, CA, USA). The effect of MK-571 on the clonal dynamics was analyzed by a mixture of 7500 cells (50% UWB1.289-pWPXL-tdTomato and 50% Olres-UWB1.289-pWPXL-EGFP) seeded per well of a 96-well black plate (costar^®^; Corning Incorporated, 3603) and treated one day later (d1) with 4 µM olaparib or the combination of 4 µM olaparib and 20 µM MK-571. The composition was analyzed using the CeligoS Image Cytometer (Nexcelom Bioscience Ltd, Lawrence, MA, USA) on the day of seeding (d0), d3 and d7. For limiting dilution experiments, the UWB1.289-pWPXL-tdTomato cells were mixed with Olres-UWB1.289-pWPXL-EGFP cells (day 0) in a ratio of 95:5%; 99:1%, 99.2:0.8%, 99.4:0.6%, 99.9%:0.1%, 99.98:0.02% and cultured in a 96-well black plate (costar^®^; Corning Incorporated, 3603). The next day, the cells were treated with 4 µM Olaparib (day 1). The culture composition was monitored every two to three days until day 29 using the CeligoS Image Cytometer (Nexcelom Bioscience Ltd, Lawrence, MA, USA).

## 3. Results

### 3.1. In Vitro Modelling of PARPi-Resistant Ovarian Cancer

We used an isogenic pair of *BRCA1*-deficient (HR-deficient) UWB1.289 vs. *BRCA1*-proficient (HR-proficient) UWB1.289+BRCA1 ovarian cancer cells (herein referred to as “UWB” and “UWB+BRCA1”) for constructing an in vitro model of PARPi resistance. UWB1.289 cells had been derived from a patient with serous ovarian cancer with a pathogenic loss-of-function mutation in *BRCA1* (2594delC) and the absence of *BRCA1* wild-type transcripts [32], which we reconfirmed in a representative early cell passage (Supplementary Table S1), and a deletion of the wild type allele, resulting in *BRCA1* loss-of-function HR-deficiency. In isogenic UWB+*BRCA1* cells, *BRCA1* had been restored by stable transfection [32,33]. According to the concept of synthetic lethality, first of all, we confirmed that UWB1.289 cells were intrinsically more sensitive to olaparib treatment compared to isogenic UWB1.289+BRCA1 cells (IC_50_ = 0.690 µM vs. IC_50_ = 3.558 µM, *p* = 0.036; Supplementary Figure S1).

**Figure 1:**
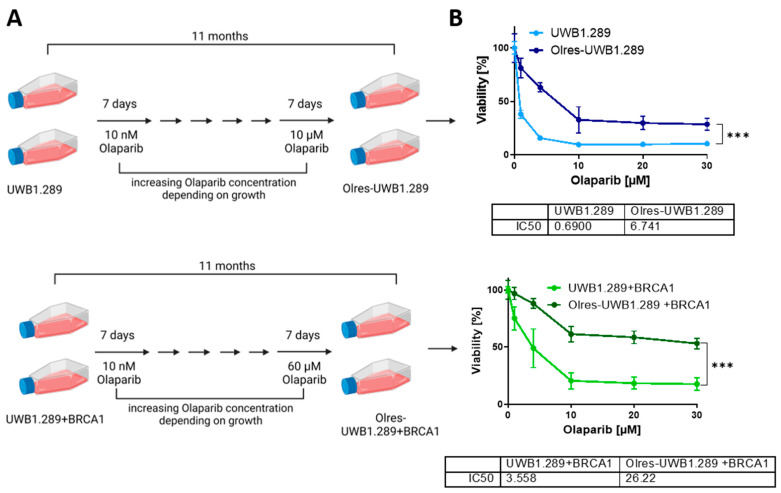
Establishment and readout of an in vitro model of PARPi-resistant ovarian cancer. (**A**) Schematic protocol for the generation of PARPi-resistant derivatives of UWB1.289 and UWB1.289+BRCA1 ovarian cancer cells by long-term exposure to incrementally ascending concentrations of olaparib. Scheme has been created with biorender.com. (**B**) Dose-response curves of olaparib treated PARPi-resistant cells according to photometric 6 d cell viability assay; IC_50_ values were determined by non-linear regression of normalized drug response; *p*-value levels according to nested *t*-test of dose-response curves are indicated; *** *p* < 0.0001.

We sought to generate an in vitro model with distinct states of olaparib resistance originating from ovarian cancer cells with a background of either HR-deficiency or HR-proficiency. Therefore, both parental cell lines were subjected to long-term olaparib exposure for 11 months in order to select for heterogenous cell clones with intrinsic or acquired olaparib resistance on cell population level (Figure 1A).

**Figure 2:**
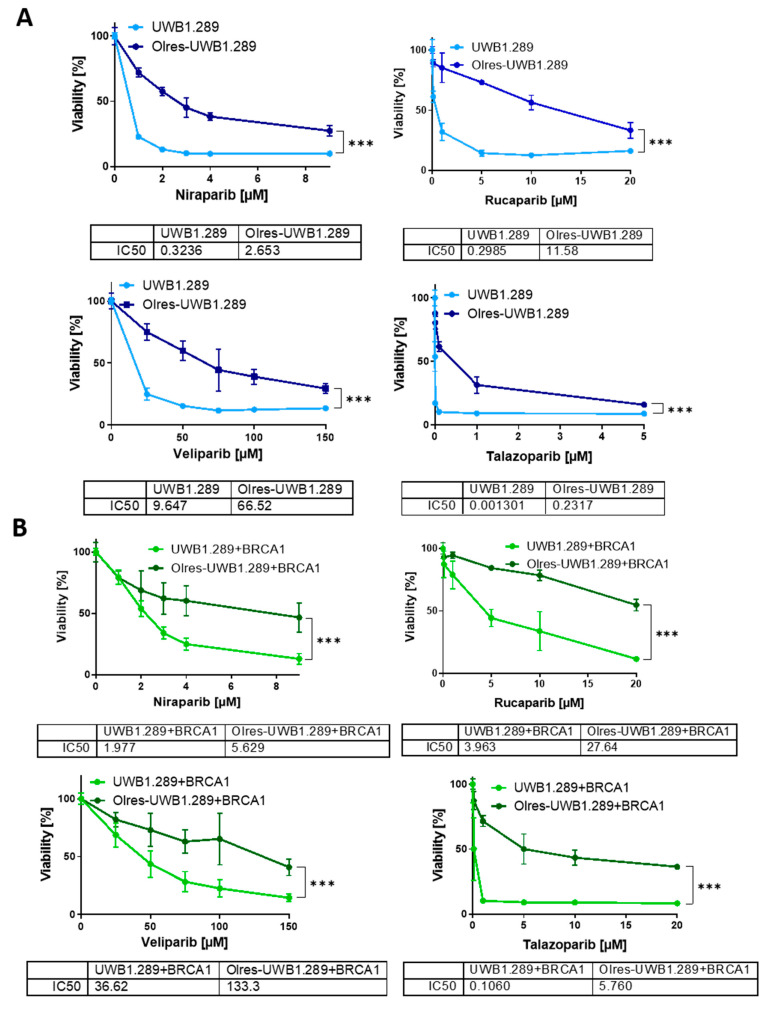
Cross-resistance spectrum of olaparib-resistant cell line to alternative PARPis. Photometric 6 d viability assays of (**A**) UWB1.289 vs. Olres-UWB1.289 and (**B**) UWB1.289+BRCA1 vs. Olres-UWB1.289+BRCA1 cells treated with niraparib, rucaparib, veliparib, or talazoparib. IC_50_ values were determined by non-linear regression of normalized drug response; *p*-value levels according to nested *t*-test of dose-response curves are indicated; *** *p* < 0.0001.

After long-term exposure to incrementally ascending olaparib concentrations up to a maintenance concentration of 10 µM, we confirmed by default 6-day viability assays that UWB cells, now termed “Olres-UWB”, gained a 9.8-fold relative resistance toward olaparib compared to parental UWB1.289 cells (UWB: IC_50_ = 0.690 µM vs. Olres-UWB: IC_50_ = 6.741 µM, *p* < 0.0001; Figure 1B). Due to their lower intrinsic response to olaparib, drug exposure of UWB+BRCA1 cells could be escalated at the same time to a maintenance concentration of 60 µM, resulting in 7.4-fold resistant “Olres-UWB+BRCA1” cells (Figure 1B, UWB+BRCA1: IC_50_ = 3.558 µM vs. Olres-UWB+BRCA1: IC_50_ = 26.22 µM, *p* < 0.0001). Notably, PARPi resistance was non-detectable by short-term 48 h viability assays (with escalated olaparib treatment up to 2000 µM), indicating that the mechanisms conferring short- term olaparib cytotoxicity have not been altered by our resistance breeding (Supplementary Figure S2). The acquired phenotype of PARPi resistance of both cell lines (Olres-UWB/Olres-UWB+BRCA1) was stable and did not change during cultivation in a drug-free medium up to four months (Supplementary Figure S3). We also observed that both Olres-UWB and Olres-UWB+BRCA1 cells exhibited a high bandwidth of cross-resistance to other well-studied PARPis, such as niraparib, rucaparib, veliparib or talazoparib (Figure 2A,B), indicating that the acquired PARPi resistance was neither unique to olaparib nor contingent on the HR-status of the parental cells.

**Figure 3:**
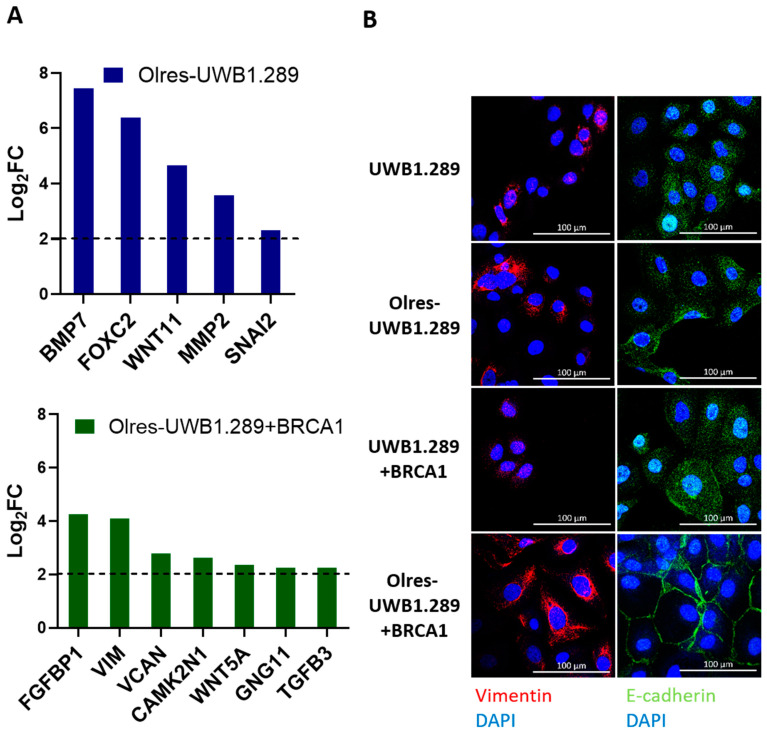
Differential expression of EMT-markers in PARPi-resistant cells. (**A**) Differential expression of EMT-associated transcripts in Olres-UWB1.289 or Olres-UWB+BRCA1 compared to the respective PARPi-sensitive parental cell lines with a log2-fold change (log_2_FC) > 2. (**B**) Differential protein expression (immunofluorescence) of vimentin or E-cadherin in Olres-UWB1.289±BRCA1 and Olres-UWB1.289±BRCA1 cells compared to the respective PARPi-sensitive parental cell lines. The scale bar depicts 100 µm.

We were further interested in the phenotypic and molecular traits of PARPi-resistant cells. *BRCA1*-transcripts were strongly upregulated in Olres-UWB+BRCA1. In Olres-UWB, however, the loss-of-function mutation in *BRCA1* (2594delC) was still detectable, and wild-type *BRCA1*-transcripts were absent, excluding that these cells restored BRCA1 function by reversion of this particular mutation (Supplementary Table S1). Under drug-free conditions, the cell cycle distribution of PARPi-resistant cells did not substantially differ from that of parental cells. Upon olaparib treatment, we observed an increase in the sub-G1 fraction of PARPi-sensitive cells, indicative of apoptosis induction [34]. However, no comparable sub-G1 increase was observed in olaparib-treated PARPi-resistant cells of both origins, clearly indicating their reduced apoptotic response to this drug (Supplementary Figure S4). Using RNA-seq data, we profiled the expression of selected EMT-related genes in PARPi-resistant cells (Supplementary Table S2). We observed upregulation of EMT-associated transcripts (e.g., *BMB7*, *FOXC2* or *SNAI2* in Olres-UWB cells/*FGFBP1*, *VIM* or *VCAN* in Olres-UWB+BRCA1; Figure 3A), with a concomitant upregulation of vimentin protein expression in both PARPi-resistant cell lines (Figure 3B).

### 3.2. PARPi-Resistant Cells Are Cross-Resistant toward Platinum-Based Chemotherapy and Exhibit a Reduced Susceptibility toward DNA Platination

**Figure 4:**
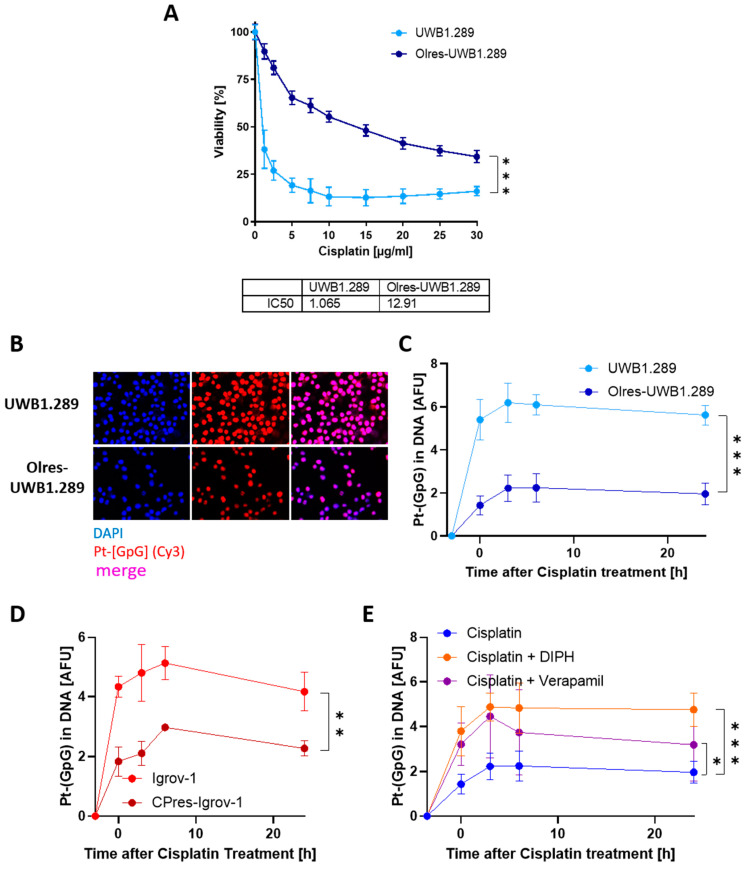
Susceptibility of PARPi-resistant cells to DNA platination and its modulation by drug efflux pump inhibitors. (**A**) Dose-response curve of cisplatin-treated PARPi-resistant cells according to fluorometric 48 h viability assay; IC_50_ values were determined by non-linear regression of normalized drug response; *p*-value levels according to nested *t*-test of dose-response curves are indicated; *** *p* < 0.0001. (**B**) Immunocytochemical staining of individual nuclei of UWB1.289 or Olres-UWB1.289 cells with DAPI and a platinum-(GpG) adduct [Pt-(GpG)] specific antibody (representative images). The images were taken with 63× magnification. Pt-(GpG) level kinetics in (**C**) UWB1.289 vs. Olres-UWB1.289 or (**D**) Igrov-1 vs. CPres-Igrov-1 cells treated with 20 µg/mL cisplatin for 3 h. Pt-(GpG) readouts after 3 h, 6 h, and 24 h after treatment are indicated; *p*-value level according to nested *t*-test of dose-response curves are indicated; *** *p* < 0.0001 ** *p* < 0.01. (**E**) Pt-(GpG) level kinetics in Olres-UWB1.289 cells with 20 µg/mL cisplatin monotreatment vs. combined treatment with 20 µg/mL cisplatin and 40 µg/mL diphenhydramine (or 50 µM verapamil); *p*-value level according to nested *t*-test of Pt-(GpG) curves are indicated *** *p* < 0.0001; * *p* < 0.05.

PARPi-resistant ovarian cancer cells exhibited a profound cisplatin cross-resistance, which was not contingent on the HR-status of the parental cell lines (Figure 4A, Supplementary Figure S5A). The cytotoxic effect of platinum-based chemotherapy is mediated by distinct DNA lesions, such as the formation of DNA-platinum adducts, also referred to as DNA platination [35]. We were further interested in how the susceptibility toward DNA platination is altered in PARPi-resistant cells. We quantified the formation of DNA-platinum adducts upon cisplatin exposure on the level of individual cell nuclei using an antibody specific for platinum-(GpG) adducts in DNA [28]. PARPi resistance in our model was associated with a strongly reduced susceptibility toward DNA platination upon exposure to cisplatin. Accordingly, a single pulse of high-dose cisplatin (20 µg/mL for 3 h) induced significantly lesser amounts of DNA platinum adducts in Olres-UWB±BRCA1 cells as compared to PARPi-sensitive parental cells (Figure 4B,C, Supplementary Figure S5B). We subsequently compared the magnitude of altered DNA platination in PARPi-resistant cells with those of a platinum-resistant reference model, represented by an isogenic pair of platinum-sensitive vs. -resistant Igrov-1 ovarian cancer cells, which we have established and characterized previously [28]. Interestingly, platinum-resistant CPres-Igrov-1 cells showed a comparably reduced susceptibility towards DNA platination than PARPi-resistant UWB cells that had never been exposed to selection pressure with any platinum drug in our hands (Figure 4D).

The level of DNA platination in cisplatin-treated cells could be indicative for the balance between cellular cisplatin import and its compensatory export via drug efflux pumps, such as MRP1, which is typically overexpressed in chemotherapy-resistant ovarian cancer [36]. Reduced susceptibility toward DNA platination in Olres-UWB±BRCA1 cells could efficiently be restored by co-treatment of cisplatin and verapamil, a pharmacological inhibitor of MDR1 (Figure 4E, Supplementary Figure S5C). A comparable or even stronger effect was observed by co-treatment with diphenhydramine (DIPH), an anti-histaminic drug that we had previously reported to inhibit the ABC-transporters MRP2, 3 and 5 [28] (Figure 4E, Supplementary Figure S5C).

To sum up, we showed that PARPi-resistant cells exhibit a profound phenotype of cisplatin cross-resistance with a concomitantly reduced susceptibility toward DNA platination upon cisplatin exposure, which was not contingent on the HR-status of the parental cells. Since DNA platination could efficiently be restored by pharmacological drug efflux inhibitors, we propose that an increased cisplatin efflux rate contributes to cisplatin cross-resistance in PARPi-resistant cells, a condition that is compatible with a phenotype of MDR.

### 3.3. PARPi-Resistant Cells Exhibit a Multidrug Resistant Phenotype with a Selective Spectrum of Cross-Resistance toward Chemotherapeutic Drugs

Based on the profound cross-resistance of PARPi-resistant cells toward cisplatin (Figure 4A), we analyzed the effect of alternative chemotherapeutic drugs on these cells, including paclitaxel, doxorubicin, pegylated liposomal doxorubicin (Caelyx), topotecan, treosulfan, and an active metabolite of irinotecan (SN-38; Supplementary Figure S6). We observed cross-resistance of Olres-UWB and Olres-UWB+BRCA1 cells toward doxorubicin and topotecan. Differential response of PARPi-resistant cells was observed for treosulfan and Caelyx: While Ores-UWB cells were cross-resistant to treosulfan, the response of Olres-UWB+BRCA1 cells toward treosulfan remained unaltered. For Caelyx, Olres-UWB cells showed similar sensitivity compared to parental cells, whereas Olres-UWB+BRCA1 had a slightly increased sensitivity to this drug compared to UWB+BRCA1 cells. No cross-resistance was observed for paclitaxel and SN-38.

We were further interested in the molecular determinants of drug resistance in PARPi-resistant cells and analyzed the activity of drug efflux transporters. Therefore, we used EFLUXX-ID^®^ green, a primary non-fluorescent marker that readily passes the cell membrane. In the cell, this marker is metabolized to a green fluorescent dye, which can only be exported by ABC-transporters. Consequently, the efflux rate of this marker mirrors ABC-transporter activity, which can be modulated with specific inhibitors (verapamil, MK-571, or novobiocin), resulting in the calculation of a “MDR activity factor” (MAF) for an ABC-transporter of interest, such as MDR1 (ABCB1), MRP1/2 (ABCC1/2) or BCRP (ABCG2).

**Figure 5:**
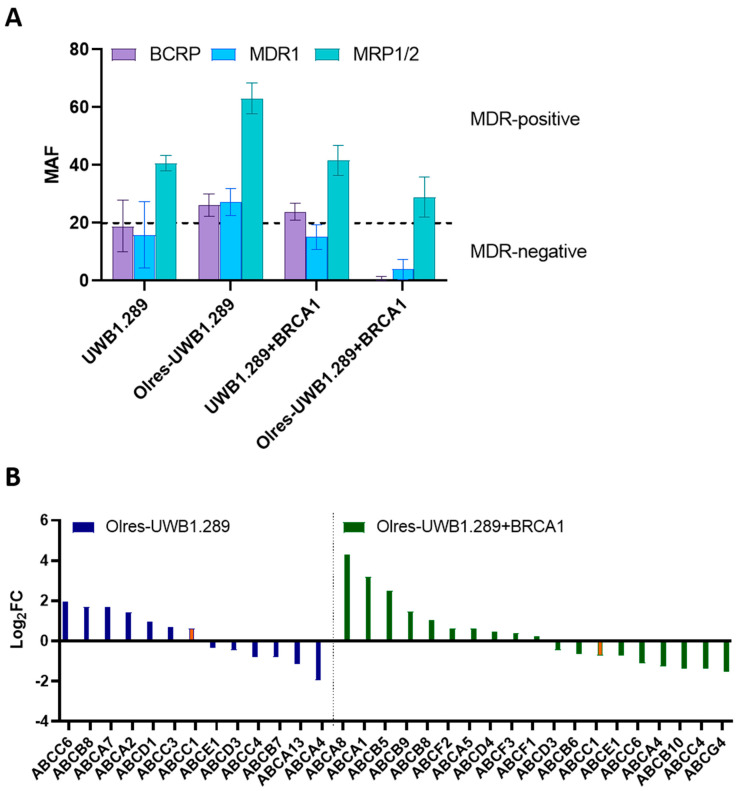
Multidrug resistance spectrum of PARPi-resistant cells. (**A**) Multidrug resistance protein activity of the ABC-transporters BCRP, MRP1/2, and MDR1 in PARPi-resistant cell lines and their respective parental PARPi-sensitive cell lines. The *y*-axis shows the multidrug-resistant activity factor (MAF) for each indicated transporter protein. A MAF score of >20 (dashed line) indicated a respective ABC-transporter activity level, which was compatible with a multidrug resistance phenotype. (**B**) Spectrum of differentially expressed transcripts of the ABC-transporter family (*p* < 0.05) in either Olres-UWB1.289 cells (left panel) or Olres-UWB1.289+BRCA1 cells compared to their respective PARPi-sensitive parental cell lines. The *ABCC1*-transcript (coding for the MRP1 protein) is indicated in orange and was functionally analyzed in (**A**).

Parental UWB cells exhibited an intrinsic level of MRP1/2 activity. However, they further converted towards MDR upon acquired PARPi resistance (Olres-UWB), associated with additional BCRP and MDR1 activity and a further increase in MRP1/2 activity (Figure 5A). Consequently, we observed mild upregulation of several ABC-transporters in Olres-UWB cells on transcript level, such as *ABCC1* (coding for MRP1; log_2_FC = 0.61), *ABCC6* (log_2_FC = 1.96), or *ABCB8* (log_2_FC = 1.70; Figure 5B; Supplementary Table S3).

Different results were obtained for the UWB+BRCA1 model. Parental cells exhibited a certain intrinsic MDR activity for BCRP and MRP1/2. Upon acquiring PARPi resistance, BCRP activity was completely lost, and MRP1/2 activity decreased. This finding was consistent with concomitant RNA-seq data since Olres-UWB+BRCA1 cells showed a completely different spectrum of ABC-transporter expression compared to Olres-UWB. While *ABCC1* was downregulated, (coding for MRP1; log_2_FC = −0.71), particularly *ABCA8* (log_2_FC = 4.32) and *ABCA1* (log_2_FC = 3.20), were strongly upregulated (Figure 5B; Supplementary Table S3).

Conclusively, we report that parental cell lines already exhibited a certain intrinsic level of MDR activity. Upon acquiring PARPi resistance, both models further converted toward MDR with co-evolved cross-resistance to other PARPis and cisplatin. However, the signature of ABC-transporter expression, their activity, and the cross-resistance spectrum toward chemotherapeutic drugs considerably diverged between the BRCA1-proficient vs. -deficient background.

### 3.4. PARP Trapping Activity in PARPi-Resistant Cells

**Figure 6:**
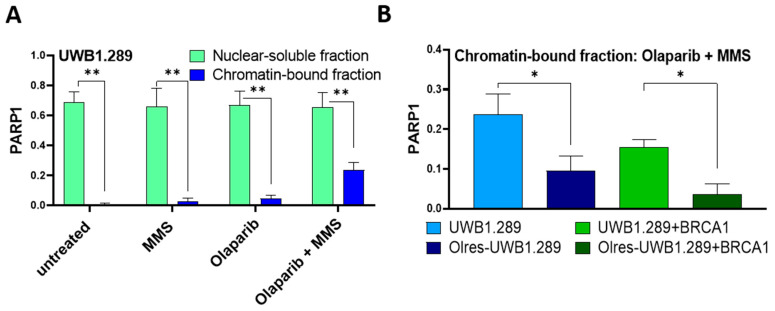
PARP trapping capacity of PARPi-resistant cells. (**A**) PARP1 protein level in the nuclear-soluble fraction vs. chromatin-bound fraction of UWB1.289 cells. (**B**) PARP1 protein level in the chromatin-bound fraction of UWB1.289 vs. Olres-UWB1.289 and UWB1.289+BRCA1 vs. Olres-UWB1.289+BRCA1 cells treated with a combination of 10 µM Olaparib and 0.01% MMS. Histone H3 and topoisomerase I levels were used as normalizing control for chromatin and nuclear soluble fractions, respectively. Additionally, PARP1 level in the chromatin fraction was corrected for contaminating topoisomerase I level. *p*-values according to the one-way ANOVA with post-hoc Tukey HSD test are indicated; * *p* < 0.05; ** *p* < 0.01.

It has been widely accepted that the cytotoxicity of PARPi, besides inhibition of DNA repair, is mediated by the ability of PARPi to stabilize PARP1-DNA complexes at single-strand breaks, a phenomenon referred to as “PARP1-trapping” [37]. We were wondering whether PARP1-trapping activity was altered in Olres-UWB cells. Therefore, we treated PARPi-resistant and parental UWB cells with olaparib ± the alkylating agent methyl methanesulfonate (MMS), which induces genotoxic DNA lesions, resulting in PARP1 activation. Finally, treated cells were separated into nuclear-soluble and chromatin-bound fractions. Under drug-free control conditions, PARP1 cells were mostly associated with the nuclear-soluble fraction (Figure 6A; Supplementary Figure S7). While olaparib alone did not alter the level of chromatin-bound PARP1 in both cell lines, combined treatment with olaparib and MMS induced an accumulation of chromatin-bound PARP1, indicating genotoxic PARP-trapping lesions, which is a surrogate for PARP1-trapping activity [37] (Figure 6A). We finally compared PARP1-trapping activity across PARPi-sensitive vs. PARPi-resistant cells treated with 10 µM olaparib and 0.01% MMS. A statistically significant reduction of PARP1-trapping activity was observed in both PARPi-resistant cell lines (Figure 6B).

Conclusively, we show that the phenotype of PARPi resistance was shaped by reduced PARP1-trapping activity, which was not contingent on the *BRCA1*-status of the parental cells.

### 3.5. Clonal Dynamics of PARPi-Resistant Cells

**Figure 7:**
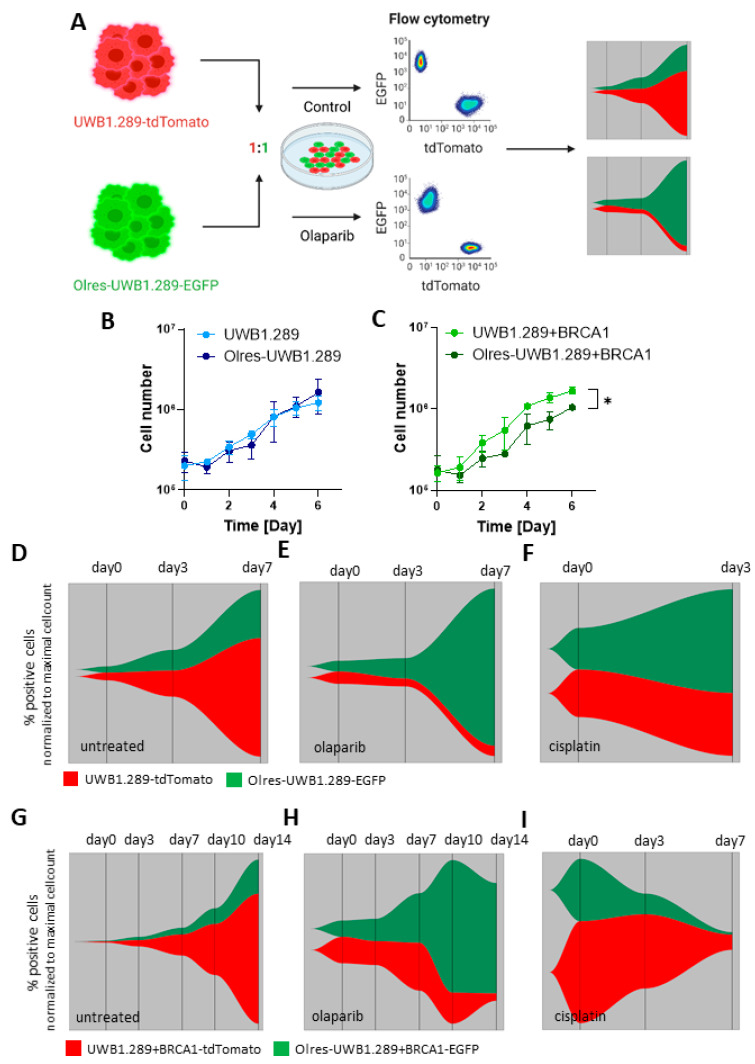

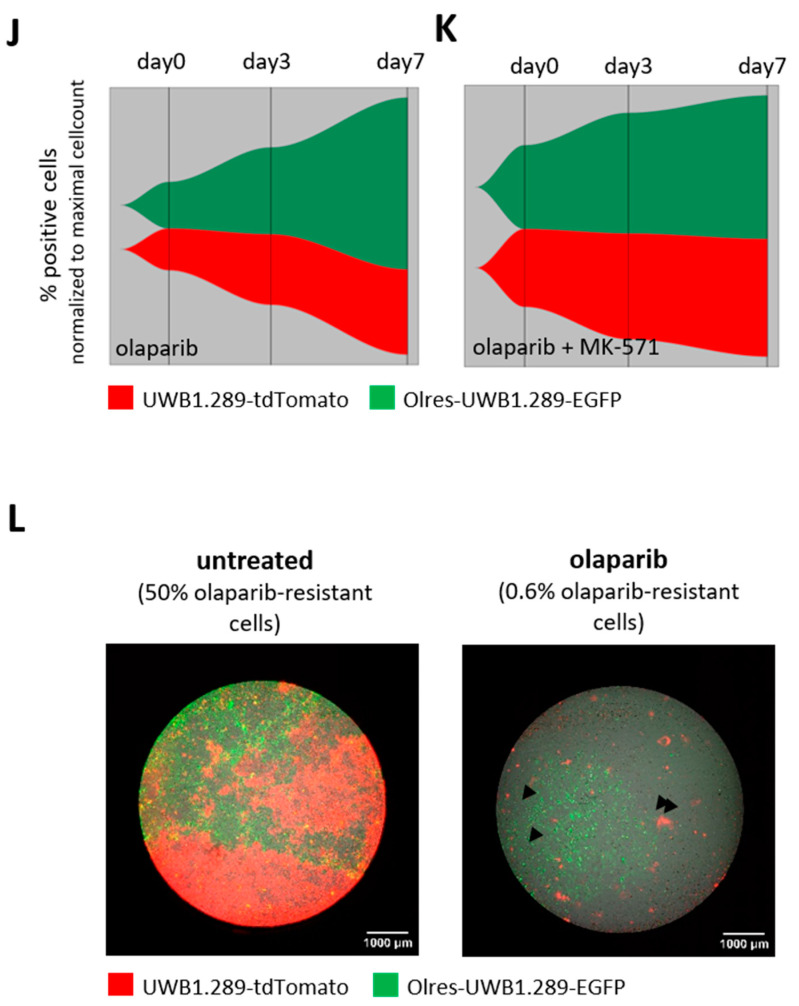
Proliferation and clonal dynamics of PARPi-resistant cells. (**A**) Schematic overview of a dual-fluorescence co-culture experiment to exemplify clonal competition of eGFP-labeled PARPi-resistant vs. tdTomato-labelled PARPi-sensitive UWB±BRCA1 cells in the presence or absence of drugs. Part of the scheme has been created with biorender.com. Growth curves showing basal proliferation of (**B**) UWB1.289 and (**C**) Olres-UWB1.289 cells in drug-free medium. *p*-values according to the nested *t*-test are indicated; * *p* < 0.05. Fish plots illustrating clonal competition of eGFP-Olres-UWB1.289 vs. tdTomato-UWB1.289 cells in the presence of (**D**) drug-free medium (**E**) 4 µM olaparib or (**F**) 1.25 µg/mL cisplatin. Fish plots illustrating clonal competition of eGFP-Olres-UWB1.289+BRCA1 vs. tdTomato-UWB1.289+BRCA1 cells in the presence of (**G**) drug-free medium (**H**) 50 µM olaparib or (**I**) 2.5 µg/mL cisplatin. Fish plots illustrating clonal competition of Olres-UWB1.289-EGFP vs. UWB1.289-tdTomato cells in the presence of (**J**) 4 µM olaparib or (**K**) 4 µM olaparib + 20 µM MK-571. (**L**) Clonal competition of Olres-UWB1.289-EGFP and UWB1.289-tdTomato seeded at a ratio of 1:1 (untreated) or 1:165 (olaparib treated). Fluorescent images were obtained 29 d after seeding. Arrowheads indicate persistent olaparib-sensitive UWB1.289-tdTomato cells.

We were finally interested in the clonal dynamics of PARPi-resistant cells under drug-free conditions or in the presence of olaparib selection pressure in a co-culture setting. Using lentiviral transduction, we color-coded PARPi-resistant (Olres-UWB or Olres-UWB+BRCA1) and PARPi-sensitive cells (UWB or UWB+BRCA1) cells with a green (eGFP) or red (tdTomato) fluorescent marker protein, respectively. After seeding PARPi-resistant and sensitive cell lines at a ratio of 1:1 in a co-culture, defining two “clones” in our model, we longitudinally tracked their cellular competition by flow cytometry for 7 d in the presence or absence of olaparib selection pressure (Figure 7A). To exclude experimental bias of this dual-fluorescent model, we first confirmed that stable expression of neither eGFP nor tdTomato provided any competitive advantage (greater than 6%, on average) to each of the studied cell line in the given observation time (Supplementary Figure S8A).

There was no significant difference in basal proliferation between PARPi-resistant Olres-UWB vs. parental PARPi-sensitive cells in monocultures (Figure 7B). Proliferation of OlresUWB+BRCA1, however, was slightly lower compared to sensitive parental cells (*p* = 0.042; Figure 7C). In the co-culture setting, we observed that Olres-UWB cells had a clear competitive disadvantage compared to PARPi-sensitive UWB cells (Figure 7D). In the presence of olaparib selection pressure, however, clonal dynamics dramatically changed, and Olres-UWB cells gained strong clonal dominance (Figure 7E). After 7 d, an almost complete “clonal sweep” was observed, driven by the expansion of PARPi-resistant cells. Interestingly, under cisplatin selection pressure, we noticed a comparable clonal expansion of PARPi-resistant cells, which was consistent with their platinum cross-resistance, as shown previously (Figure 4A and Figure 7F).

We subsequently repeated these experiments for the UWB+BRCA1/Olres-UWB+BRCA1 cells. We observed similar clonal dynamics in this model, including a competitive disadvantage of Olres-UWB+BRCA1 under drug-free conditions and their clonal dominance in the presence of olaparib or cisplatin monotreatment (Figure 7G–I). Notably, clonal shifts in this model were principally slower than in UWB/Olres-UWB cell lines, consistent with the fact that HR-proficient cells are principally less responsive to olaparib. This prompted us to extend the observation time from 7 to 14 days in this assay. All results, reported herein, were successfully reproduced when swapping the color-code between the respective cell lines (Supplementary Figure S8B), which excludes an experimental bias of the color-coding procedure.

Particularly for the UWB/Olres-UWB model, we were interested in how the clonal dynamics of PARPi-resistant cells could be pharmacologically modulated, e.g., by co-treatment with olaparib-sensitizing drugs. Consistent with the confirmed MDR phenotype of Olres-UWB and the activity of the MRP1/2 transporter (Figure 5), we observed that the strong clonal selection of Olres-UWB under olaparib treatment could be mitigated by co-treatment with the MRP1 inhibitor MK-571, exemplifying a potential PARPi-sensitization strategy on the level of clonal dynamics (Figure 7J–K).

Finally, we performed limiting dilution experiments and discovered that not more than 0.6% PARPi-resistant cells, co-cultured at a ratio of 1:165 with 96.4% PARPi sensitive cells (30 Olres-UWB co-cultured with 4970 UWB cells in a 96-well scale) were sufficient for an almost complete “clonal sweep” of Olres-UWB cells (Figure 7L). Interestingly, we observed that single PARPi-sensitive cells occasionally persisted under olaparib treatment, indicating that these cells may already have had intrinsic resistance to the PARPi (Figure 7L, arrowheads).

Taken together, we report that our in vitro model of PARPi resistance mirrors common clonal dynamics of therapy resistance. While PARPi-resistant cells show a clear “fitness penalty” of their resistant phenotype under drug-free conditions, they gained strong clonal dominance under olaparib selection pressure and progressively replaced PARPi-sensitive cells. This is a proof-of-principle that our model allows studying the effect of experimental platinum-sensitizers on PARPi-resistant cells on the level of clonal dynamics, herein exemplified by the MRP1 inhibitor MK-571.

## 4. Discussion

In the present study, we successfully established an in vitro model of PARPi resistance, allowing direct comparison of resistance phenotypes derived from a HR-proficient vs. HR-deficient isogenic background. According to the standard clinical care, ovarian cancer patients with HRD are currently treated with a combination of bevacizumab and PARPi olaparib [5]. Therefore, not only pathways of PARPi resistance but also pathways of bevacizumab resistance may contribute to recurrent disease. Since in vitro modeling of dual resistance (PARPi and bevacizumab) is highly challenging and difficult to interpret, we focused with our experimental design only on PARPi resistance, allowing us a clear dissection of different resistance mechanisms specific to PARPi.

Our model is principally in line with previous studies in experimental models of ovarian cancer, reporting on the emergence of an acquired phenotype of PARPi resistance after long-term PARPi exposure [17,19,20,21]. Still, one previous study reported that olaparib exposure is unlikely to produce an acquired resistance phenotype [22]. This could be due to a completely different schedule of olaparib exposure in this study, with several cycles of 48 h treatment followed by drug-free recovery after each cycle. This suggests that the applied dosage and timing of long-term olaparib exposure may govern the nature of the induced resistance phenotype [21] and areimportant variables to consider. Moreover, the final assessment of PARPi sensitivity by dose-response curves was performed in this study after a considerably shorter time of olaparib treatment (72 h vs. 6 d) compared to our study. Olaparib resistance in our model was only detectable by viability assays after 6 d olaparib exposure and not after short-term exposure of 48 h. This suggests that the mechanisms of short-term olaparib cytotoxicity have not been altered in our PARPi-resistant cells and points to the relevance of olaparib exposure times for detecting a PARPi-resistant phenotype.

The rigor of our model is corroborated by the following basic characteristics. Firstly, our cell lines reflect the known spectrum of cytotoxicity of the different PARPis, with talazoparib providing the strongest effect on the cells and veliparib with the weakest effect [38,39]. Secondly, parental *BRCA1*-deficient cells were more sensitive to olaparib compared to *BRCA1*-proficient cells, consistent with the concept of synthetic lethality [40]. Accordingly, the final maintenance concentration of olaparib, reached after 11 months of long-term olaparib exposure, was six times higher in Olres-UBW+BRCA1 compared to Olres-UWB cells. However, the relative gain of resistance to olaparib was roughly comparable across these two models (Olres-UWB 9.8-fold vs. Olres-UWB+BRCA1 7.4-fold). This suggests that the relative efficacy of our protocol to select for an acquired resistance to olaparib was only slightly influenced by the HR-status of the parental cells.

We revealed that PARPi-resistant cells have increased expression of the EMT-associated marker vimentin. These findings can likely be interpreted as basal cellular adaption mechanisms in response to drug exposure. Particularly EMT has already been described as an adaptive response to therapy, independent of clonal selection [41], and has been associated with intrinsic and acquired resistance to talazoparib [15]. 

The cross-resistance of PARPi-resistant cells to cisplatin was the focus of our interest since the clinical question of how to optimize post-PARPi treatment in ovarian cancer patients remains open, given the collective data that PARPi resistance is likely to be linked to a reduced response to subsequent platinum-based chemotherapy [42]. While HRD contributes to sensitivity to both platinum and PARPi [12], alterations in the nucleotide excision pathway, for instance, were shown to result in discordant responses to these drugs [43]. In our model, PARPi-resistant cell lines had a strongly impaired susceptibility to DNA platination upon cisplatin exposure, which could be restored by drug efflux pump inhibitors. This shows that long-term exposure to olaparib is likely to alter the platinum export capacity and induce MDR-associated cross-resistance toward cisplatin. We further tested this hypothesis with a functional analysis of ABC-transporters. While parental cell lines already exhibited a certain level of intrinsic MDR activity, resulting PARPi-resistant cells from both models further converted toward MDR. However, the signature and activity of ABC-transporter expression and the cross-resistance spectra to other chemotherapeutic drugs considerably diverged between the *BRCA1*-proficient vs. *BRCA1*-deficient models. In *BRCA1*-proficient cells, for instance, the activity of BCRP and MRP1/2 decreased, whereas a strong increase in *ABCA8* transcription was observed, which has already been described in the context of MDR in pancreatic cancer [44]. Although we are fully aware that MDR activity and transcript levels are not directly comparable, this suggests that the MDR phenotype is a continuum of complex molecular alterations involving the activation of a variety of ABC-transporters, which can be shaped by diverging molecular phenotypes and different intensities. Notably, a mentioned study on a murine PARPi resistance model did not show consistent patterns of cross-resistance to cisplatin and other DNA-damaging agents in cells with acquired PARPi resistance [23]. Overall, this demonstrates the need to consider cell-line-specific effects when comparing different in vitro models.

In vitro PARPi resistance has been suggested to arise from clonal selection of an intrinsically unstable heterogenous population in a generally sensitive cell line containing pre-existing PARPi-resistant cells [16]. Although Olres-UWB±BRCA1 cells have been generated on a cell population level, resulting in a mixture of molecularly heterogeneous clones of PARPi-resistant cells, we reduced this to a simplified model and defined two “clones” in our competition assay, i.e., a “PARPi-sensitive” vs. a “PARPi-resistant” clone. Although this strategy does not entirely reflect the monitoring of “true clones” that are usually defined by single nucleotide- and/or copy number variations it nonetheless allowed us to monitor population dynamics of PARPi-sensitive vs. PARPi-resistant cells.

PARPi-resistant cells readily gained clonal dominance under olaparib selection pressure, even when not more than 30 resistant cells were seeded among 4970 sensitive cells. Therefore, our approach models the principles of recurrent ovarian cancer driven by a small fraction of resistant cells under olaparib selection pressure. Interestingly, single PARPi-sensitive cells occasionally persisted under olaparib treatment, which possibly were intrinsically resistant to the PARPi. This reflects the evolution of the acquired resistance of tumors. It is consistent with the hypothesis that resistance is a “fait accompli”, indicating that the time to recurrence is simply the interval required for a resistant subclone to re-populate a lesion [45]. Notably, the fitness advantage of PARPi-resistant cells was critically dependent on the presence of olaparib as PARPi-sensitive cells slowly dominate the co-cultures under drug-free conditions. Our findings are in line with other 2D and 3D co-culture models [46,47] and reflect the “fitness penalty” of drug resistance [48]. This is particularly true as MDR cancer cells require additional energy to maintain and synthesize drug efflux proteins, thus likely “trading-off” non-essential functions, such as proliferation [49]. The clonal dominance of PARPi-resistant cells over PARPi-sensitive cells in the presence of olaparib is, moreover, consistent with the principle so-called “biased cellular competition” [50]. Thus, PARPi-resistant cells have increased fitness in the presence of olaparib compared to parental PARPi-sensitive cells since they previously have been “biased” towards an increased tolerance of olaparib by long-term exposure to this drug. 

Our in vitro platform could be used as a simplified model for a heterogenous tumor with differential PARPi sensitivity at a subclonal level. It may provide an ideal basis to monitor the dynamic evolution of PARPi-resistant cells under various treatment conditions, such as clonal sweeps of PARPi-resistant cells under olaparib selection pressure, which drive cancer progression. Moreover, our model will allow us to test whether an experimental PARPi-sensitizer, in our case exemplified by MK-571, may counteract the clonal expansion of PARPi-resistant cells under olaparib treatment.

## 5. Conclusions

We present a well-characterized in vitro model, which could be instrumental in dissecting mechanisms of PARPi resistance from HR-proficient vs. HR-deficient backgrounds and in studying the clonal dynamics of PARPi-resistant cells in response to experimental olaparib-sensitizers. Furthermore, our model could provide an ideal platform for functional analysis of PARPi resistance, e.g., by CRISPR-Cas9-mediated genomic screens.

## Data Availability

All relevant data are included into the manuscript. Raw data can be made available upon reasonable request from the senior authors. RNA-seq data have been deposited at the GEO databank (accession number pending).

## Supplementary Materials

The following supporting information can be downloaded at: https://www.mdpi.com/article/10.3390/cancers15153774/s1, Supplementary Figure S1: Synthetic lethality of olaparib in the UWB1.289 in vitro model of BRCA1-deficient ovarian cancer; Supplementary Figure S2: Response of PARPi-resistant cells to short term olaparib exposure (48 h); Supplementary Figure S3: Time-dependent stability of the PARPi-resistant phenotype in Olres-UWB1.289±BRCA1 cells; Supplementary Figure S4: Cell cycle analysis of PARPi-resistant cells; Supplementary Figure S5: Susceptibility of Olres-UWB1.289+BRCA1 cells to DNA platination and its modulation by drug efflux pump inhibitors; Supplementary Figure S6: Cross-resistance spectrum of PARPi-resistant cells; Supplementary Figure S7: PARP trapping capacity of PARPi-resistant UWB+BRCA1 cells; Supplementary Figure S8: Clonal competition of PARPi-resistant cells; Supplementary Table S1: BRCA1/2 mutational analysis of parental UWB1.289±BRCA1 cell lines and their PARPi-resistant Olres-UWB1.289±BRCA1 derivatives extracted from whole exome sequencing data; Supplementary Table S2: List of EMT-associated genes of the RT^2^ Profiler plate; Supplementary Table S3: ABC-transporter genes differentially expressed in between Olres-UWB1.289 and UWB1.289 as well as Olres-UWB1.289+BRCA1 and UWB1.289+BRCA1.
