# Supplementary material for: Establishment and Molecular Characterization of an In Vitro Model for PARPi-Resistant Ovarian Cancer
Source: Cancers (Basel). 2023 Jul 25;15(15):3774. doi: 10.3390/cancers15153774 (PMC10417418; doi:10.3390/cancers15153774)
Supplement: Supplementary file 1 [file cancers-15-03774-s001.zip › cancers-2482447-supplementary.pdf]

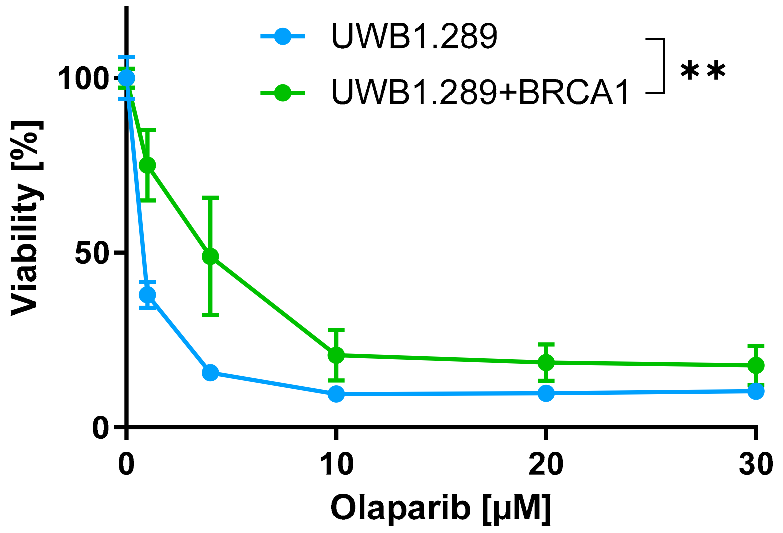

	UWB1.289	UWB1.289+BRCA1
IC50	0.6900	3.558

Supplementary Figure S1: **Synthetic lethality of olaparib in the UWB1.289 *in vitro* model of BRCA1-deficient ovarian cancer.** Dose-response curve of olaparib treated UWB1.289 *vs.* UWB1.289+BRCA1 cells according to photometric 6 d viability assay; IC_{50} values were determined by non-linear regression of normalized drug response; p-value levels according to nested t-test of dose-response curves are indicated; ** $p<0.01$.

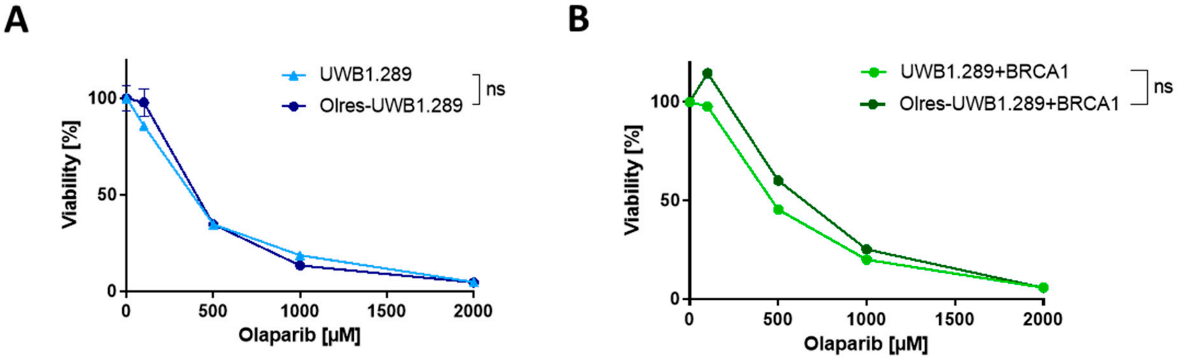

	UWB1.289	Olres-UWB1.289
IC50	292.6	316.1

	UWB1.289+BRCA1	Olres-UWB1.289+BRCA1
IC50	394.7	569.7

Supplementary Figure S2: Response of PARPi-resistant cells to short term olaparib exposure (48h). Dose-response curve of olaparib treated (**A**) UWB1.289 *vs.* OlresUWB1.289 or (**B**) UWB1.289+BRCA1 *vs.* Olres-UWB1.289+BRCA1 cells according to fluorometric 48 h viability assay; IC_{50} values were determined by non-linear regression of normalized drug response; p-value levels according to nested t-test of dose-response curves were non-significant (ns).

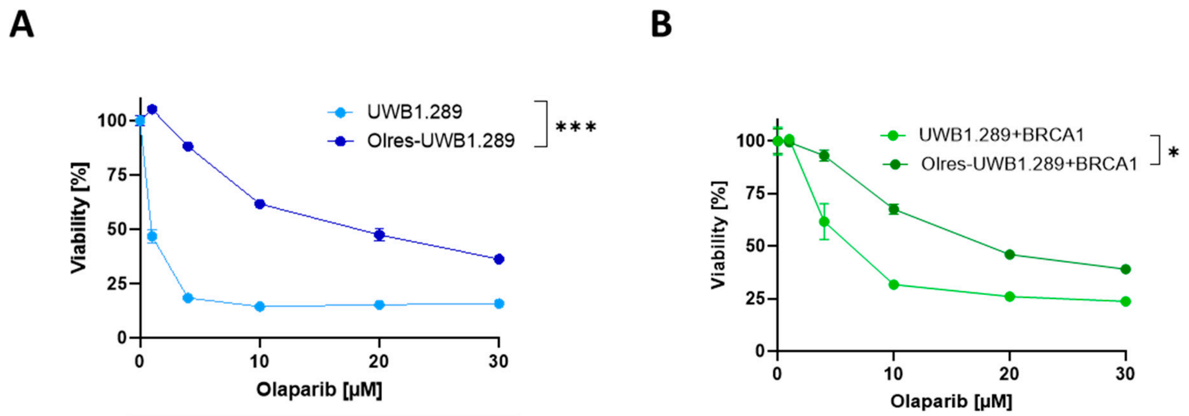

	UWB1.289	Olres-UWB1.289
IC50	1.029	18.53

	UWB1.289+BRCA1	Olres-UWB1.289+BRCA1
IC50	6.830	20.56

Supplementary Figure S3: Time-dependent stability of the PARPi-resistant phenotype in Olres-UWB1.289±BRCA1 cells. Olaparib response of (**A**) Olres-UWB1.289 and (**B**) OlresUWB1.289+BRCA1 cells after continuous cultivation in drug-free medium for 4 months. Dose-response curves were determined by photometric 6 d viability assays and are shown in comparison to respective PARPi-sensitive parental cells (UWB1.289 or UWB1.289+BRCA1, respectively). IC_{50} values were determined by non-linear regression of normalized drug response; p-value levels according to nested t-test of dose-response curves are indicated; *** $p<0.0001$, * $p<0.05$.

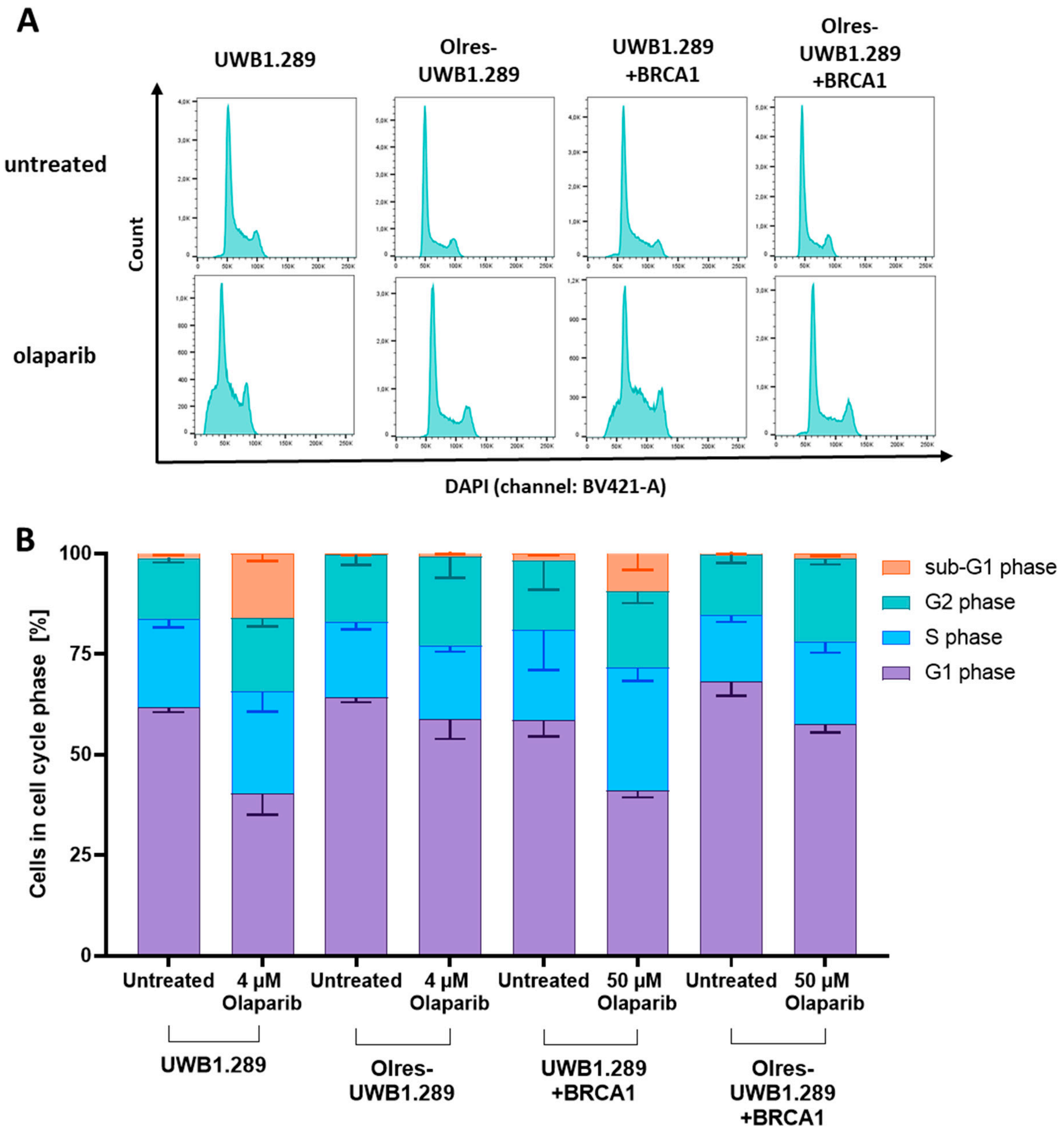

Supplementary Figure S4: Cell cycle analysis of PARPi-resistant cells. A) Representative flowcytometric results of DAPI-labeled cells following olaparib treatment (4 µM in UWB1.289 and Olres-UWB1.289; 50 µM in UWB1.289+BRCA1 and Olres-UWB1.289+BRCA1; 72 h) *vs.* incubation under drug-free conditions (72 h). **B**) Transformation of results into cell cycle phase distribution of cell lines under the above-mentioned experimental conditions.

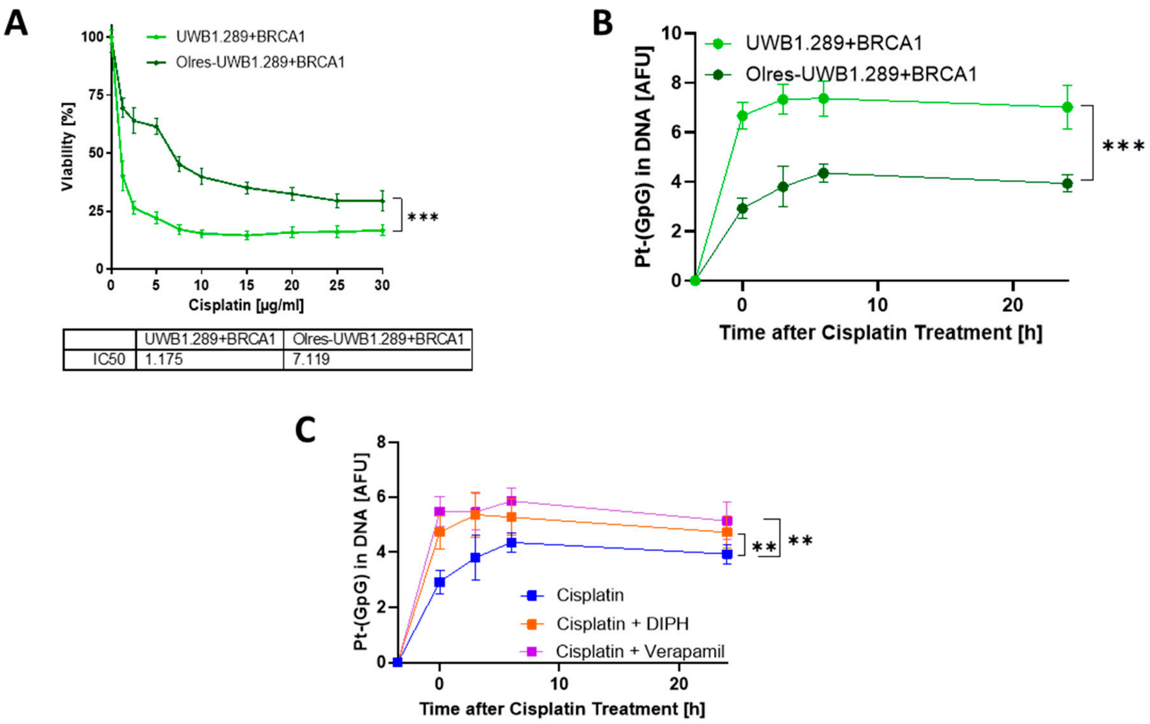

	UWB1.289+BRCA1	Olres-UWB1.289+BRCA1
IC50	1.175	7.119

Supplementary Figure S5: Susceptibility of Olres-UWB1.289+BRCA1 cells to DNA platination and its modulation by drug efflux pump inhibitors. A) Dose-response curve of cisplatin treated Olres-UWB1.289+BRCA1 cells according to fluorometric 48 h viability assay; IC_{50} values were determined by non-linear regression of normalized drug response; p-value levels according to nested t-test of dose-response curves are indicated; *** p<0.0001. **B**) Platinum-DNA adduct [Pt-(GpG)] level kinetics in UWB1.289+BRCA1 *vs.* Olres-UWB1.289+BRCA1. Pt-(GpG) readouts after. 3h. 6h and 24h after treatment are indicated; p-value level according to nested t-test of dose-response curves are indicated; *** p<0.0001 ** p<0.01. **C**) Pt-(GpG) level kinetics in Olres-UWB1.289+BRCA1 cells with 20µg/ml cisplatin monotreatment *vs.* combined treatment with 20µg/ml cisplatin and 40µg/ml diphenhydramine (or 50 µM verapamil); p-value level according to nested t-test of Pt-(GpG) curves are indicated ** p<0.01.

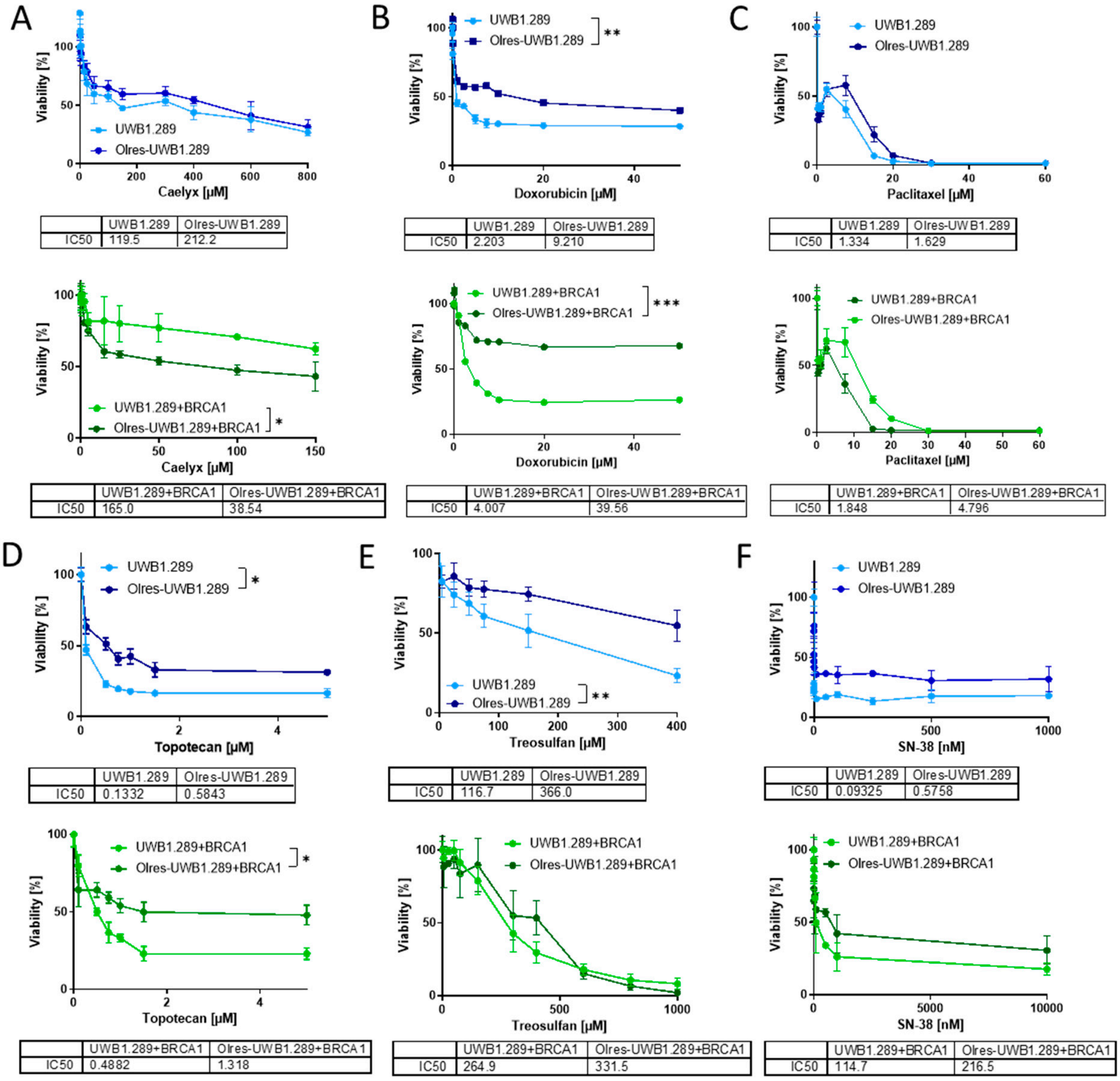

Supplementary Figure S6: Cross-resistance spectrum of PARPi-resistant cells. Dose-response curve of UWB1.289/OlresUWB1.289 or UWB1.289+BRCA1/Olres-UWB1.289+BRCA1 treated with (**A**) Caelyx (**B**) doxorubicin (**C**) paclitaxel (**D**) topotecan (**E**) treosulfan and (**F**) SN-38 according to fluorometric 48 h viability assay; IC_{50} values were determined by non-linear regression of normalized drug response; p-value levels according to nested t-test of dose-response curves are indicated; * p<0.05, ** p<0.01, *** p<0.0001.

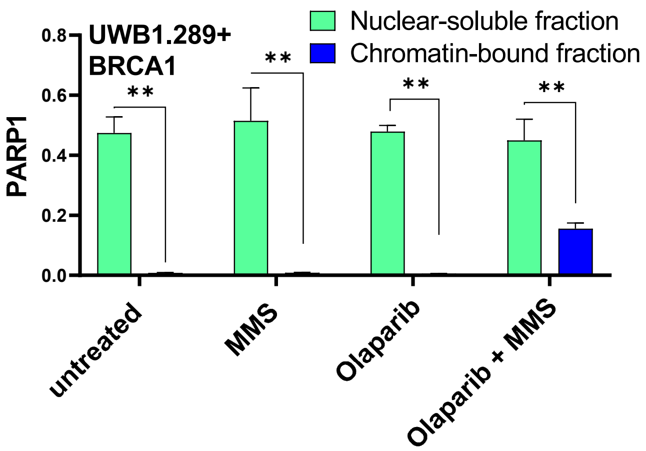

Supplementary Figure S7: PARP trapping capacity of PARPi-resistant UWB+BRCA1 cells. PARP1 protein level in nuclear-soluble fraction and chromatin-bound fraction of UWB1.289+BRCA1 cells. Significance between the nuclear-soluble and the chromatin-bound fraction was calculated by one-way ANOVA with post-hoc Tukey HSD test; ** $p<0.01$.

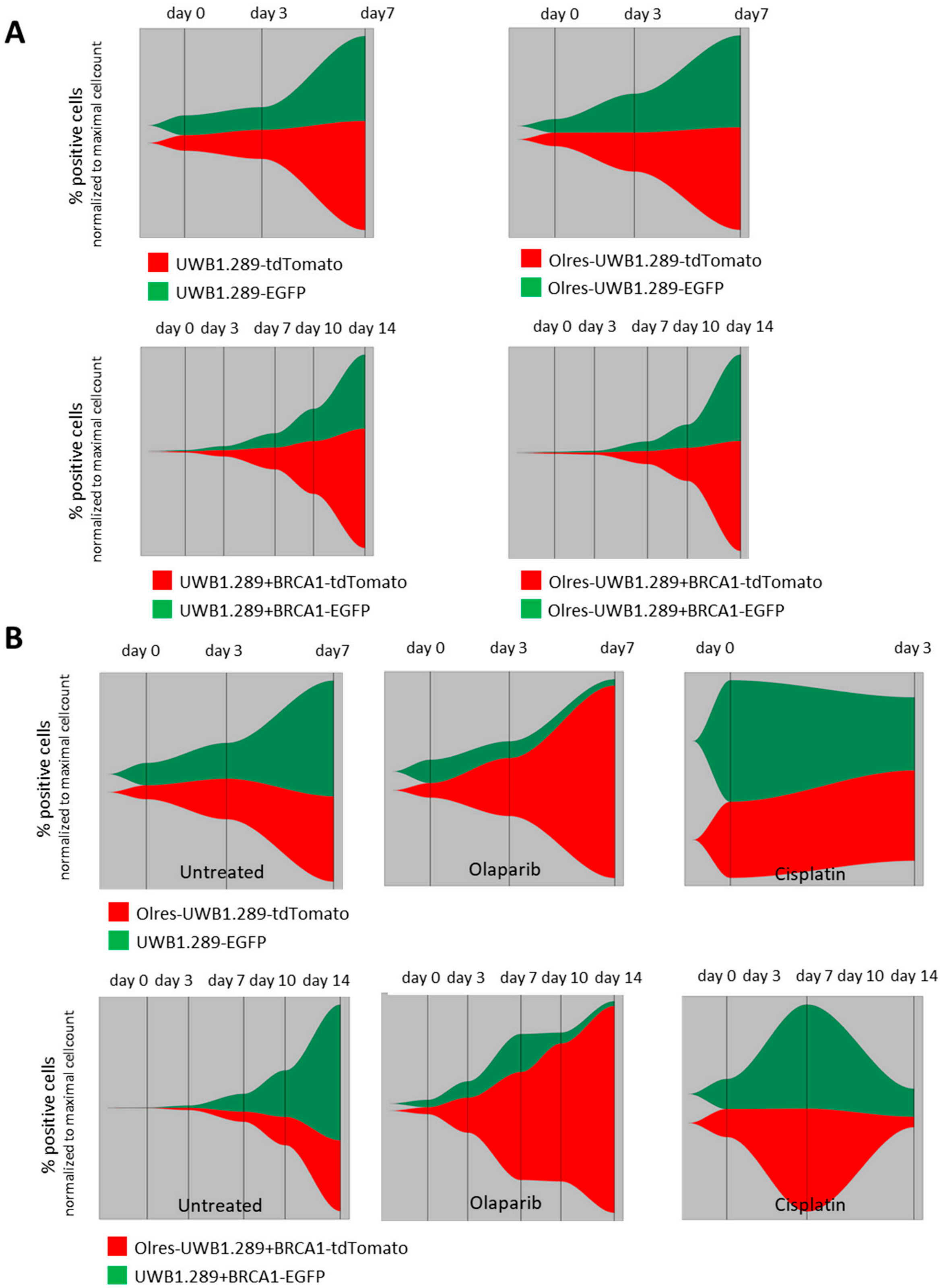

Supplementary Figure S8: Clonal competition of PARPi-resistant cells. A) Control experiments showing if lentiviral eGFP- *vs*. tdTomato labelling within one cell line confers competitive advantages. Therefore, each cell line was labeled with eGFP and tdTomato and was transferred to a co-culture system at a ratio of 1:1 under drug-free conditions. Resulting fishplots allow to exclude, that either eGFP or tdTomato confer a significant competitive advantage to the each of the four respective cell lines of the *in vitro* model. B) Control experiment in the context of the clonal dynamics of PAPRi-

resistant cells. The experimental setting has been designed similar to **Figure 6D-I** in the main text with the exception that the color-code between the respective PARPi-resistant cells and their parental PARPi-sensitive counterparts has been swapped. Fishplots are shown, illustrating clonal competition of tdTomato-Olres-UWB1.289 vs. eGFP-UWB1.289 cells in the presence of drug-free medium, 4µM olaparib or 1.25µg/ml cisplatin.

Supplementary Table S1: BRCA1/2 mutational analysis of parental UWB1.289±BRCA1 cell lines and their PARPi-resistant Olres-UWB1.289±BRCA1 derivatives extracted from whole exome sequencing data.

UWB1.289

Chr	Region	Type	Reference	Allele	Gene	Transcript	Nucleotide Change	Amino Acid Change	Count	Coverage	Frequency
13	32906729	SNV	A	C	BRCA2	NM_000059.3	c.1114A>C	p.Asn372His	81	149	54.36
13	32929387	SNV	T	C	BRCA2	NM_000059.3	c.7397T>C	p.Val2466Ala	63	63	100.00
17	41245073	Deletion	G	-	BRCA1	NM_007300.3	**c.2475delC**	p.Asp825fs	52	54	96.30

Olres-UWB1.289

Chr	Region	Type	Reference	Allele	Gene	Transcript	Nucleotide Change	Amino Acid Change	Count	Coverage	Frequency
13	32906729	SNV	A	C	BRCA2	NM_000059.3	c.1114A>C	p.Asn372His	71	140	50.71
13	32929387	SNV	T	C	BRCA2	NM_000059.3	c.7397T>C	p.Val2466Ala	84	84	100.00
17	41245073	Deletion	G	-	BRCA1	NM_007300.3	**c.2475delC**	p.Asp825fs	81	81	100.00

UWB1.289+BRCA1

Chr	Region	Type	Reference	Allele	Gene	Transcript	Nucleotide Change	Amino Acid Change	Count	Coverage	Frequency
13	32906729	SNV	A	C	BRCA2	NM_000059.3	c.1114A>C	p.Asn372His	70	146	47.95
13	32929387	SNV	T	C	BRCA2	NM_000059.3	c.7397T>C	p.Val2466Ala	74	74	100.00
17	41223094	SNV	T	C	BRCA1	NM_007300.3	c.4900A>G	p.Ser1634Gly	26	75	34.67
17	41244000	SNV	T	C	BRCA1	NM_007300.3	c.3548A>G	p.Lys1183Arg	31	105	29.52
17	41244435	SNV	T	C	BRCA1	NM_007300.3	c.3113A>G	p.Glu1038Gly	23	111	20.72
17	41244936	SNV	G	A	BRCA1	NM_007300.3	c.2612C>T	p.Pro871Leu	21	78	26.92
17	41245073	Deletion	G	-	BRCA1	NM_007300.3	**c.2475delC**	p.Asp825fs	53	91	58.24

Olres-UWB1.289+BRCA1

Chr	Region	Type	Reference	Allele	Gene	Transcript	Nucleotide Change	Amino Acid Change	Count	Coverage	Frequency
13	32906729	SNV	A	C	BRCA2	NM_000059.3	c.1114A>C	p.Asn372His	36	59	60.02
13	32929387	SNV	T	C	BRCA2	NM_000059.3	c.7397T>C	p.Val2466Ala	32	32	100.00
17	41223094	SNV	T	C	BRCA1	NM_007300.3	c.4900A>G	p.Ser1634Gly	19	78	24.36
17	41244000	SNV	T	C	BRCA1	NM_007300.3	c.3548A>G	p.Lys1183Arg	33	103	32.04
17	41244435	SNV	T	C	BRCA1	NM_007300.3	c.3113A>G	p.Glu1038Gly	23	91	25.27
17	41244936	SNV	G	A	BRCA1	NM_007300.3	c.2612C>T	p.Pro871Leu	18	71	25.35
17	41245073	Deletion	G	-	BRCA1	NM_007300.3	**c.2475delC**	p.Asp825fs	44	70	62.86

Supplementary Table S2: List of EMT-associated genes of the RT^2 Profiler plate. These genes were used to identify EMT-associated genes differentially expressed between Olres-UWB1.289 and UWB1.289 as well as Olres-UWB1.289+BRCA1 and UWB1.289+BRCA1.

Epithelial to Mesenchymal Transition (EMT)

AHNAK AKT1 BMP1 BMP2 BMP7 CALD1 CAMK2N1 CAV2 CDH1 CDH2 COL1A2 COL3A1 COL5A2 CTNNB1 DESI1 DSC2 DSP EGFR ERBB3 ESR1 F11R FGFBP1 FN1 FOXC2 FZD7 GEMIN2 GNG11 GSC GSK3B IGFBP4 IL1RN ILK ITGA5 ITGAV ITGB1 JAG1 KRT14 KRT19 KRT7 MAP1B MMP2 MMP3 MMP9 MSN MST1R NODAL NOTCH1 NUDT13 OCLN PDGFRB PLEK2 PTK2 PTP4A1 RAC1 RGS2 SERPINE1 SMAD2 SNAI1 SNAI2 SNAI3 SOX10 SPARC SPP1 STAT3 STEAP1 TCF3 TCF4 TFPI2 TGFB1 TGFB2 TGFB3 TIMP1 TMEFF1 TMEM132A TSPAN13 TWIST1 VCAN VIM VPS13A WNT11 WNT5A WNT5B ZEB1 ZEB2

Supplementary Table S3: ABC-transporter genes differentially expressed (padj<0.05) between Olres-UWB1.289 and UWB1.289 as well as Olres-UWB1.289+BRCA1 and UWB1.289+BRCA1. The log_2FC as well as p-value and adjusted p-value are shown.

	gene	log_2FC	p-value	adjusted p-value
**Upregulated genes in Olres-UWB1.289:**				
	ABCA2	1.4378183	0.00063631	0.002232757
	ABCA7	1.701753165	$7.2449{\cdot}10^{-9}$	$6.90172E{\cdot}10^{-08}$
	ABCB8	1.707356736	$1.6393{\cdot}10^{-9}$	$1.73265{\cdot}10^{-08}$
	ABCC1	0.617449164	$1.6451{\cdot}10^{-8}$	$1.47318{\cdot}10^{-07}$
	ABCC3	0.696911095	$3.4895{\cdot}10^{-8}$	$2.95847{\cdot}10^{-07}$
	ABCC6	1.965985272	0.000623	0.002190605
	ABCD1	0.98837655	0.00031914	0.001204058
**Downregulated genes in Olres-UWB1.289:**				
	ABCA13	-1.155283739	0.014513419	0.035576327
	ABCA4	-1.973320787	$9.389{\cdot}10^{-25}$	$5.50492{\cdot}10^{-23}$
	ABCB7	-0.823928937	$3.39687{\cdot}10^{-11}$	$4.70869{\cdot}10^{-10}$
	ABCC4	-0.786272441	$4.4451{\cdot}10^{-5}$	0.000205172
	ABCD3	-0.458678591	$1.46654{\cdot}10^{-5}$	$7.49021{\cdot}10^{-05}$
	ABCE1	-0.366158372	0.000441074	0.001614263
**Upregulated genes in Olres-UWB1.289+BRCA1:**				
	ABCA1	3.196342498	$3.36943{\cdot}10^{-75}$	$4.42723{\cdot}10^{-73}$
	ABCA5	0.622378971	0.000642471	0.002047219
	ABCA8	4.319236953	0.002145555	0.006140393
	ABCB5	2.503372263	$1.34791{\cdot}10^{-14}$	$1.47682{\cdot}10^{-13}$
	ABCB8	1.06603417	0.000392591	0.001297218
	ABCB9	1.468924647	0.01274436	0.030725352
	ABCD4	0.450386668	0.001798519	0.005245253
	ABCF1	0.25067348	0.007548075	0.019206839
	ABCF2	0.623741406	0.002916781	0.008122776
	ABCF3	0.376386358	0.00203143	0.005852677
**Downregulated genes in Olres-UWB1.289+BRCA1:**				
	ABCA4	-1.258707187	$5.74{\cdot}10^{-49}$	$3.48{\cdot}10^{-47}$
	ABCB10	-1.364263057	$8.60687{\cdot}10^{-16}$	$1.03306{\cdot}10^{-14}$
	ABCB6	-0.657261409	0.009037341	0.02261813
	ABCC1	-0.715748239	$2.54222{\cdot}10^{-15}$	$2.94932{\cdot}10^{-14}$
	ABCC4	-1.367320159	$3.00849{\cdot}10^{-11}$	$2.45203{\cdot}10^{-10}$
	ABCC6	-1.108749245	0.01027885	0.025384648
	ABCD3	-0.45131147	$2.27724{\cdot}10^{-06}$	$1.07796{\cdot}10^{-05}$
	ABCE1	-0.741485537	$6.62831{\cdot}10^{-14}$	$6.8511{\cdot}10^{-13}$
	ABCG4	-1.546262453	0.000275741	0.000938102
